# Does health economics research align with the disease burden in the Middle East and North Africa region? A systematic review of economic evaluation studies on public health interventions

**DOI:** 10.1186/s41256-022-00258-y

**Published:** 2022-07-25

**Authors:** Mouaddh Abdulmalik Nagi, Mustafa Ali Ali Rezq, Sermsiri Sangroongruangsri, Montarat Thavorncharoensap, Pramitha Esha Nirmala Dewi

**Affiliations:** 1 Doctor of Philosophy Program in Social, Economic and Administrative Pharmacy, Department of Pharmacy, Faculty of Pharmacy, Mahidol University, Bangkok, Thailand; 2 Faculty of Medical Sciences, Aljanad University for Science and Technology, Taiz, Yemen; 3 Master of Public Health, Department of Social and Preventive Medicine, Faculty of Medicine, University of Malaya, Kuala Lumpur, Malaysia; 4 Faculty of Pharmacy, Sana’a University, Sana’a, Yemen; 5 Social and Administrative Pharmacy Excellence Research (SAPER) Unit, Department of Pharmacy, Faculty of Pharmacy, Mahidol University, Bangkok, 10400 Thailand; 6 Department of Pharmacy Profession, Faculty of Medicine and Health Sciences, Universitas Muhammadiyah Yogyakarta, Yogyakarta, Indonesia

**Keywords:** Cost-effectiveness, Economic evaluation, Middle East, North Africa, Public health

## Abstract

**Introduction:**

Economic evaluation studies demonstrate the value of money in health interventions and enhance the efficiency of the healthcare system. Therefore, this study reviews published economic evaluation studies of public health interventions from 26 Middle East and North Africa (MENA) countries and examines whether they addressed the region's major health problems.

**Methods:**

PubMed and Scopus were utilized to search for relevant articles published up to June 26, 2021. The reviewers independently selected studies, extracted data, and assessed the quality of studies using the Consolidated Health Economic Evaluation Reporting Standards (CHEERS) checklist.

**Results:**

The search identified 61 studies. Approximately half (28 studies; 46%) were conducted in Israel and Iran. The main areas of interest for economic evaluation studies were infectious diseases (21 studies; 34%), cancers (13 studies; 21%), and genetic disorders (nine studies; 15%). Five (8%), 39 (64%), 16 (26%), and one (2%) studies were classified as excellent, high, average, and poor quality, respectively. The mean of CHEERS checklist items reported was 80.8% (SD 14%). Reporting the structure and justification of the selected model was missed in 21 studies (37%), while price and conversion rates and the analytical methods were missed in 21 studies (34%).

**Conclusions:**

The quantity of economic evaluation studies on public health interventions in the MENA region remains low; however, the overall quality is high to excellent. There were obvious geographic gaps across countries regarding the number and quality of studies and gaps within countries concerning disease prioritization. The observed research output, however, did not reflect current and upcoming disease burden and risk factors trends in the MENA region.

## Introduction

The purposes of health interventions are not only to diagnose, treat, or relieve diseases but also to prevent and protect against illnesses [1]. Interventions to provide safe drinking water, healthy food, and clean air, along with interventions to control vectors, control tobacco and alcohol, detect diseases, promote regular exercise and a healthy lifestyle, prevent injury and avoid risky behaviors, are deemed health interventions that could improve overall health, longevity, and productivity of communities [2, 3]. Despite their effectiveness in most cases, these public health interventions always need resources. With a limited health care budget, implementing such interventions must compete with other interventions for the same resources. Thus, to maximize health outcomes within a limited budget, health technology assessment (HTA), particularly health economics (HE) or economic evaluation studies which provide value for money derived from the investment, plays an important role in informing healthcare resource allocation decisions [4, 5]. However, compared to other health interventions, there is limited evidence in economic evaluation studies on public health intervention [4].

Despite its current limited role in policy decision-making, the number of HE articles published in the Middle East and North Africa (MENA) countries has recently increased and begun to attract the interest of policy-makers to inform priority-setting toward achieving universal health coverage (UHC) in many countries [6]. Similar to other regions, the growing demand for such kind of evidence in MENA is mainly driven by several factors, including rising healthcare costs as a result of remarkable advances and high costs of healthcare technologies, the World Health Organization (WHO)’s recommendations on the use of HTA, and the movement towards evidence-based healthcare system [5, 7]. Hence, in the face of the inevitable pressures from competing for alternative interventions, countries should direct their finite health budgets to meet the priority health needs of their populations to reach fair and efficient outcomes [8].

Furthermore, the burden of diseases and their background risk factors in the MENA region have shifted dramatically in the past decades, with wide variation across countries [9–11]. The region is also dealing with an epidemiological shift in burden from infectious to chronic diseases. In addition, some communicable diseases have recently re-emerged [12]. In the meantime, cardiovascular diseases (CVDs), cancers, diabetes mellitus, chronic kidney diseases, and chronic lung diseases represent a significant disease burden in the MENA region and pose a growing threat to public health [13]. These diseases impose enormous pressures on the health system and resources. Moreover, this burden increases with alarming future prevalence projections of up to 2.4 million deaths from the diseases mentioned earlier in the region in 2025 [14]. Coupled with ongoing wars and turmoil, several risk factors affect the people’s health in this region [9]. In this regard, MENA has some of the highest rates of non-communicable diseases (NCDs)-risk factors, such as high blood pressure, obesity, physical inactivity, tobacco use, and high intake of salt, sugar and fats, along with the absence of an efficient surveillance system for early-stage disease detection in most countries [15]. Therefore, MENA countries need integral strategies built on existing expertise and projects to handle the existing health challenges and the ones that may occur in the future.

Based on this background, this study aims to systematically review the characteristics and critically assess the quality of economic evaluation studies on public health interventions in MENA countries. It also aims to examine whether the economic evaluation studies have addressed the major health problems and are suitable for policy decision-making in the region. The findings of this study may support existing evidence for better allocating healthcare and research resources in the region.

## Methods

This review was reported following the Preferred Reporting Items for Systematic Review and Meta-analyses (PRISMA) statement [16].

### Data sources and search strategy

The researchers searched the literature in PubMed and Scopus for relevant articles published since databases inception up to June 26, 2021. The search strategy involved combining terms for public health interventions and economic evaluations using the Boolean ‘AND’, ‘OR’, and ‘NOT’ operators. The term "public health" was purposefully broad, and the authors expected that it would incorporate a wide array of interventions in the field. Search terms used involved the combination of the following Medical Subject Heading (MeSH) terms and keywords: (health promotion [MeSH]) OR (public health intervention [Title/Abstract]) OR (Exercise [MeSH]) OR (smoking cessation [MeSH]) OR (Mass Screening [MeSH]) OR (prevention [Title/Abstract]) OR (tobacco control [Title/Abstract]) OR (Public Policy [MeSH]) AND (cost [MeSH]) OR (Cost–Benefit Analysis [MeSH]) OR (economic evaluation [Title/Abstract]) AND (Middle East [MeSH]) OR (North Africa [MeSH]) OR (Djibouti [Title/Abstract]) OR (Mauritania [Title/Abstract]) OR (Pakistan [Title/Abstract]) OR (Palestine [Title/Abstract]) OR (Sudan [Title/Abstract]) OR (Somalia [Title/Abstract]) NOT (vaccine [MeSH]) OR (vaccination [MeSH]) OR (immunization [MeSH]) OR (immunization program [Title/Abstract]) OR (immunoprophylaxis [Title/Abstract]). The search was restricted to journal articles, studies of human subjects, and studies written in the English language.

### Eligibility criteria

Economic evaluation studies (cost–benefit analyses, cost-effectiveness analyses, cost-minimization analyses, and cost-utility analyses) of public health interventions available in full text, published in English, and about at least one country in the MENA region were included in this review. In this study, the researchers adopted the comprehensive definition of the MENA region [17], which included 24 countries (i.e., Afghanistan, Algeria, Bahrain, Djibouti, Egypt, Iran, Iraq, Jordan, Kuwait, Lebanon, Libya, Mauritania, Morocco, Oman, Pakistan, Palestine, Qatar, Saudi Arabia, Somalia, Sudan, Syria, Tunisia, United Arab Emirates, and Yemen). Besides, Turkey and Israel were added to the analysis as they were involved in PubMed (MeSH) definition of the Middle East region. Meanwhile, cost of illness studies and other partial economic evaluation studies were excluded. The researchers also excluded the full economic evaluation studies on vaccines (unless screening was one of the comparators) and diagnostic and therapeutic interventions. These interventions were reviewed separately in other manuscripts to ensure harmony and consistency of compared studies, thus, gaining more insight into the specific characteristics of each group of interventions [18, 19].

### Study selection and data extraction

Study selection was performed independently by two reviewers (MAN and SS) according to the exclusion and inclusion criteria; first by title, followed by abstract, and finally by full-text screening. Discrepancies were resolved by discussion and consensus with another reviewer (MT). Then, identified studies were screened independently by two reviewers (MAN and MAAR) to extract (1) general information (e.g., year and country of publication, author’s affiliation, disease/risk factor domain, type of analysis, the model used, and interventions and comparators assessed); (2) methodological characteristics (e.g., the year of estimation, study population, perspective, time horizon, the discount rate, model’s internal validity, double-counting, and the type of sensitivity analysis performed); (3) source of input parameters (e.g., epidemiology, effectiveness, utility, and cost data). In addition, the source of funding, result and conclusion, cost-effectiveness threshold, and types of included costs were also extracted. Any disagreements were resolved in discussion with another reviewer (MT).

### Quality assessment and appraisal

The methodological and reporting quality of each included economic evaluation study was assessed by two independent reviewers (MAN, PEND). Disagreement was resolved by discussion and consensus with the third reviewer (MT). Studies were assessed for their quality of reporting by their compliance with the Consolidated Health Economic Evaluation Reporting Standards (CHEERS) statement [20]. Studies were then scored against each of the applicable 24 checklist items according to whether reporting was fully satisfied or did not satisfy the item requirements. Each reporting quality criterion was rated as reported (score = +), not reported if the item was missing when expected to be reported (score = −) or not applicable (NA). Subsequently, studies were classified by their percentage of applicable items as "excellent", "high", "average", or "poor" quality when they fulfilled 100%, 76–99%, 50–75%, or less than 50% of the CHEERS checklist items, respectively [18]. The overall quality of reporting was presented as a percentage score of applicable items. Studies scoring above 75% were considered of higher reporting quality.

### Data management and analysis

EndNote X8 software was utilized for duplicate removal. All data extracted from the final list of included studies were entered into a pre-designed data extraction form in Microsoft Excel 2013 spreadsheet. Data synthesis involved stratifying and summarizing the evidence by intervention type, appraising the economic evaluation methods used to assess interventions, and presenting the cost-effectiveness outcomes. Then, descriptive statistics were used with percentages, mean, standard deviation (SD), and median to display the results of extracted data and evaluated items obtained in this review.

## Results

### Search results

**Fig. 1:**
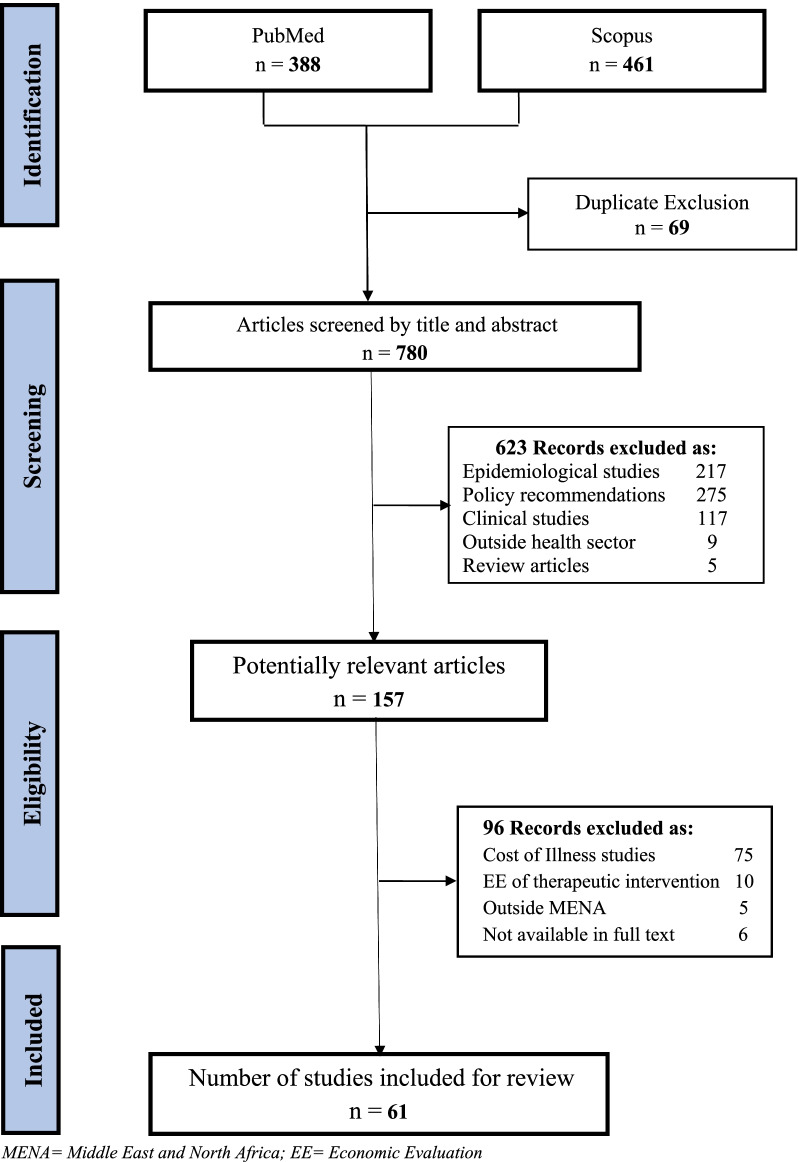
PRISMA flow chart of search procedure

In total, 849 studies were identified following the initial search (i.e., 388 from PubMed and 461 from Scopus). The removal of 69 duplicates resulted in 780 remaining articles. Title and abstract screening resulted in the removal of 623 papers and left 157 potentially relevant studies for full article text screening. Finally, 61 studies [21–81] were included in this review. The reasons for exclusion in each step are provided in the PRISMA flowchart in Fig. 1.

### General and methodological characteristics

This review identified economic evaluation studies on screening programs (36 studies; 59%) [21, 22, 24, 26, 28–31, 33–37, 39, 41, 45, 48–52, 54–56, 58, 60, 63–65, 70, 71, 73, 74, 79–81] and health promotion campaigns (four studies; 7%) [40, 46, 59, 78]. In addition, two studies concerned vector control programs [43, 68] and two studies for smoking cessation interventions [57, 66] (3% each), while the remaining 17 studies (28%) focused on other different interventions, as shown in Table 1. The main areas of interest for economic evaluation studies identified in this review were infectious diseases (21 studies; 34%) [21, 22, 25, 28–31, 33, 38, 39, 43, 44, 50, 55, 61, 62, 67, 68, 72, 75, 76], cancers (13 studies; 21%) [26, 34, 35, 37, 41, 45, 51, 54, 60, 63, 69, 71, 73], genetic disorders (nine studies; 15%) [36, 48, 49, 52, 64, 65, 70, 74, 79], CVDs (seven studies; 12%) [24, 40, 46, 59, 78, 80, 81], gestational diabetes mellitus (two studies; 3.3%) [56, 58], maternal issues (two studies; 3.3%) [27, 77], tobacco control (two studies; 3.3%) [57, 66], and other domains (five studies; 8%) [23, 32, 42, 47, 53]. Regionally, all disease domains that accounted for the highest burden (i.e., CVDs, cancers, diabetes, chronic kidney and lung diseases, and nutritional diseases and injuries) had not been adequately studied or even not studied at all in some MENA countries. Similarly, the most prevalent risk factors (smoking, physical inactivity, obesity, high blood pressure, and high intake of salt, sugar and fats) were not studied in almost all MENA countries.

**Table 1:** General characteristics of the included studies

Author (references)	Country	Publication year	Affiliation	Domain	Type of study	Model used	Intervention and comparator
Adibi et al. [21]	Iran	2004	National	Infectious	CEA (per infection averted)	Decision tree	HBsAg screening for all premarriage individuals and prevention protocol for seronegative subjects or HBsAg screening for all premarriage individuals, HBcAb screening in the HBsAg negative spouses of the HBsAg positive persons, and prevention protocol Vs no screening and no prevention
Al Abri et al. [22]	Oman	2020	National and International	Infectious	CUA (per QALY)	Decision tree and Markov	Different testing programs using an IGRA versus the TST, combined with QFT-Plus with 6H, QFT-Plus with 3HP, QFT-Plus with 4R, TST with 6H, TST with 3HP and directly observed therapy, TST with 4R, Vs CXR alone
Al‐Qudah et al. [23]	Jordan	2019	National and International	NCDs	CBA	NA	Clinical pharmacist intervention (home medication management review, patient education on drug-drug interactions, dosage adjustment, patient education on the importance of adherence to their medication regime, etc.) Vs routine care
Assanelli et al. [24]	Algeria and others	2015	National and International	CVDs	CEA (per LYG)	NR	ECG in combination with family and personal history and physical examination (no comparator was reported)
Balicer et al. [25]	Israel	2005	National	Infectious	CBA	NA	3 strategies for the use of stockpiled antiviral drugs during a pandemic: (1) therapeutic use, (2) long-term preexposure prophylaxis, and (3) short-term postexposure prophylaxis for close contacts of influenza patients
Barfar et al. [26]	Iran	2014	National	Cancer	CEA (per case detected)	NR	Mammography breast cancer screening Vs no screening
Carvalho et al. [27]	Afghanistan	2013	National and International	Maternal diseases	CEA (per LYS)	Decision tree	Family planning strategies Vs combined several interventions (Integrated reproductive health and pregnancy-related services)
Chodick et al. [28]	Israel	2005	National	Infectious	CEA (per case avoided)	Decision tree and Markov	Mass Varicella zoster virus vaccination, screening followed by vaccination, vaccination of carriers and do nothing (status quo situation)
Chodick et al. [29]	Israel	2002	National	Infectious	CUA (per QALY)	Decision tree and Markov	Mass Hepatitis A vaccination, screening and vaccination Vs passive immunization (status quo strategy)
Chowers [30]	Israel	2017	National	Infectious	CUA (per QALY)	Decision tree and Markov	Universal prenatal HIV screening compared with the current high risk only screening policy
Devine [31]	Afghanistan and others	2020	International	Infectious	CUA (per DALY)	Decision tree	Gender-based treatment according to qualitative G6PD rapid diagnostic screening Vs routine care
El-Dahiyat [32]	Jordan	2017	National	All	CMA	NA	Generic medicines Vs originator brand medicines
El-Ghitany [33]	Egypt	2019	National	Infectious	CEA (per test performed)	NR	EGCRISC use Vs mass screening
Eltabbakh et al. [34]	Egypt	2015	National	Cancer	CUA (per QALY)	NR	HCC screening program by ultrasound and alpha-fetoprotein Vs diagnosis outside the program
Gamaoun et al. [35]	Tunisia	2018	National	Cancer	CEA (per case avoided)	Decision tree	Two-dose HPV vaccine for young girls Vs screening with three time-lapse Pap smear test
Ginsberg et al. [36]	Israel	1998	National	Genetic disorders	CBA	NA	Combined educational and national prenatal screening programs for thalassemia by blood samples test then electrophoresis for samples with abnormal values followed by counselling Vs no screening
Ginsberg et al. [37]	Israel	2013	National	Cancer	CUA (per DALY)	Markov	Screening with removal of cancerous lesions, Three doses of HPV vaccine with or without booster dose every 20 years for 12-year old girls, treatment of all cancer cases, and combinations of these interventions in different scenarios Vs current policy (Treat all cases and annual screening of 12.1% of females aged 12–70 with Pap smear)
Ginsberg et al. [38]	Israel	2020	National	Infectious	CUA (per DALY)	Decision tree	Continuous HIV pre-exposure prophylaxis regimen Vs on-demand HIV pre-exposure prophylaxis
Ginsberg et al. [39]	Israel	2007	National	Infectious	CUA (per QALY)	Markov	Screening with Pap smear (annually, tri-annually and penta-annually), HPV-DNA testing, or Visual inspection with acetic acid (VIA) -All followed by removal of cancerous lesions-, prevention by vaccination (3 doses of HPV vaccine with or without booster dose every 10 years for 12-year old girls), treatment of all cancer cases, screening and treatment, vaccination and treatment, and combination of prevention, screening and treatment. All strategies Vs current policy (all cases are treated and 12.2% of females aged 12–70 receive Pap smear)
Ginsberg et al. [40]	Israel	2012	National	CVDs	CUA (per DALY)	NR	Home, school, workplace, restaurant and supermarket-based interventions, screening, media strategies, taxation of unhealthful food products, provision of government subsidies to reduce the price of healthful foods [and vice-versa], mandatory food labeling, and prohibiting the sale of unhealthful foods in vending machines
Haghighat et al. [41]	Iran	2016	National and International	Cancer	CUA (per QALY)	Decision tree and Markov	Mammography screening strategy Vs no screening
Hamdani et al. [42]	Pakistan	2020	National and International	Mental diseases	CEA (per unit change in anxiety, depression and functioning scores)	NR	Problem Management + Vs enhanced usual care
Howard et al. [43]	Pakistan	2017	National and International	Infectious	CUA (per DALY), CEA (per LYG, case prevented, death prevented)	NR	Vector control using annual indoor residual spraying Vs routine malaria diagnosis and treatment
Hussain et al. [44]	Pakistan	2019	International	Infectious	CUA (per DALY), CEA (per patient treated)	Decision tree and Markov	Active case finding program using incentives Vs the existing passive case finding and treatment program
Javadinasab et al. [45]	Iran	2017	National	Cancer	CUA (per QALY) and CEA (per LYG)	Markov	Colonoscopy screening every 5 years starting at age 40, screening every 10 years starting at age 40, screening every 5 years starting at age 50, screening every 10 years starting at age 50, screening once/lifetime at age 50, and screening once/lifetime at age 55 Vs no screening
Javanbakht [46]	Iran	2018	National and International	CVDs	CEA (per capita healthcare cost)	Markov	Adequate dairy foods consumption Vs inadequate dairy foods consumption
Kashi et al. [47]	Pakistan and others	2019	International	Malnutrition	CUA (per DALY)	Decision tree	Multiple micronutrient supplementation Vs iron and folic acid supplementation
Khneisser et al. [48]	Lebanon	2015	National	Genetic disorders	CBA	NA	Expanded newborn screening for inborn errors of metabolism by using tandem mass spectrometry followed by diagnostic confirmation and management Vs clinical "late" detection
Khneisser et al. [49]	Lebanon	2007	National	Genetic disorders	CBA	NA	G6PD deficiency screening Vs no screening
Kim et al. [50]	Egypt	2015	National and International	Infectious	CUA (per QALY)	Decision tree and Markov	One-time screening and follow-up treatment for HCV infection Vs the current strategy of no screening
Kim et al. [51]	Algeria, Lebanon, and Turkey	2013	National and International	Cancer	For vaccination: CUA (per DALY). For screening: CEA (per LYS)	Decision tree	Three doses of HPV vaccine for all 12-year girls in MENA countries and combination of screening and vaccination in Algeria, Lebanon, and Turkey Vs no intervention
Koren et al. [52]	Israel	2014	National	Genetic disorders	CEA (per case prevented)	NR	Thalassemia prevention program Vs routine treatment of β Thalassemia major and its complications
Lahiri et al. [53]	MENA and others	2005	International	Back pain	CEA (per LYG)	Markov	Worker training, engineering controls coupled with administrative controls, a combination of worker training and engineering controls, and the full ergonomics program Vs no intervention
Leshno et al. [54]	Israel	2003	National	Cancer	CEA	Markov	One-time colonoscopic screening, colonoscopy followed by a 10-year follow-up, annual FOBT, annual FOBT and flexible sigmoidoscopy, and annual detection of altered human DNA in stool Vs no screening
Lim et al. [55]	Pakistan	2020	National and international	Infectious	CEA (per cured case)	Markov	HCV Screening and treatment Vs no interventions
Lohse et al. [56]	Israel and others	2011	International	DM	CUA (per DALY)	Decision tree	Gestational diabetes mellitus screening and lifestyle change Vs no intervention
Madae’en et al. [57]	Jordan	2020	National	Smoking	CEA (per LYG)	Markov	Varenicline for 3 months, NRT (combined patch and gum) for 3 months, and physician advice over three visits with no medications Vs no intervention
Marseille et al. [58]	Israel and others	2013	National and international	DM	CUA (per DALY)	Decision tree	Initial screening tests, antenatal care for Gestational DM women, and post-partum DM prevention interventions Vs no Gestational DM screening and treatment
Mason et al. [59]	Tunisia, Syria, Palestine and Turkey	2014	National and International	CVDs	CEA (per LYG)	Decision tree	Health promotion campaign, labelling of food packaging or mandatory salt reduction of processed foods Vs no policy
Messoudi et al. [60]	Morocco	2019	National and International	Cancer	CEA (per LYG)	Markov	Screening of women aged 30–49 years with a VIA test every 3 years Vs no intervention, two doses of HPV vaccine for pre-adolescent girls Vs no intervention, and combined HPV vaccine and screening Vs screening alone
Mostafa et al. [61]	Egypt	2019	National and International	Infectious	CUA (per QALY)	Decision tree and Markov	Safety-engineered syringes Vs conventional syringes
Mostafa et al. [62]	Egypt	2019	National	Infectious	CUA (per QALY)	Decision tree	Safety-engineered syringes Vs conventional syringes
Nahvijou et al. [63]	Iran	2016	National and International	Cancer	CUA (per QALY)	Markov	11 different screening strategies with different periodicities and different intervals Vs no screening
Okem et al. [64]	Turkey	2017	National	Genetic disorders	CEA (per cases detected or procedural related losses avoided)	Decision tree	For women < 35-year of age: triple test, combined test, Non-invasive Prenatal Screening Test (NIPT) by using cell free fetal DNA, NIPT as a second-step screening for high-risk patients detected by triple test, and NIPT as a second-step screening for high-risk patients detected by combined test. For women ≥ 35-year of age: implementing invasive test (amniocentesis) and NIPT for all women were compared Vs current screening strategies
Ornoy et al. [65]	Israel	2019	National	Genetic disorders	CBA	NA	National screening program for attention deficit hyperactivity disorder among school children and continue treatment until adulthood. The comparator was not reported
Ranson et al. [66]	MENA	2002	International	Smoking	CUA (per DALYs)	NR	Price increases, NRT, and a package of non-price interventions other than NRT (such as comprehensive bans on advertising and promotion, bans on smoking in public places, prominent warning labels and mass consumer information). The comparator was not reported
Rashidian et al. [67]	Iran	2015	National	Infectious	CEA (per percentages of volume reduction and weight reduction)	Decision tree	Medical waste treatment devices called Saray 1, Saray 2, Sazgar, KAZU, Newster, Ecodas T150, Ecodas T300, and Newster 10, Vs Caspian-Alborz
Rezaei-Hemami et al. [68]	Iran	2014	National	Infectious	CEA (per averted malaria case)	NR	Larviciding, indoor residual spraying, insecticide treated net, set up the diagnosis and treatment in less than 24 h, and set up the border facilities Vs each other
Saygili et al. [69]	Turkey	2019	National	Cancer	CEA (per quality of life unit and level of satisfaction)	NR	Comprehensive palliative care center, hospital inpatient services, and home healthcare services Vs each other
Shamshiri et al. [70]	Iran	2012	National and International	Genetic disorders	CUA (per DALY)	Decision tree	Congenital hypothyroidism screening programs Vs no screening
Sharma et al. [71]	Lebanon	2017	National and International	Cancer	CEA (per LYS)	Markov	Increasing cytologic screening coverage to 50% at 3 and 5 years interval Vs annual screening at 20% coverage
Shlomai et al. [72]	Israel	2020	National	Infectious	CUA (per QALY) and CEA (per death prevented)	Markov	Social distancing and national lockdown Vs complete isolation of infected individuals or individuals at high exposure risk in a dedicated facility
Shmueli et al. [73]	Israel	2013	National and International	Cancer	CUA (per QALY)	Decision tree	Low-dose computed tomography screening Vs no screening
Sladkevicius et al. [74]	Libya	2010	National and International	Genetic disorders	CEA (per LYG)	Decision tree	Neonatal screening for Phenylketonuria Vs no screening
Verguet et al. [75]	Djibouti, Mauritania, Somalia, Sudan and others	2013	International	Infectious	CUA (per DALY)	Markov	Adding HIV pre-exposure prophylaxis at pre-existing levels Vs existing HIV prevention interventions (male circumcision, antiretroviral therapy and condom use)
Vijayaraghavan et al. [76]	Afghanistan	2006	International	Infectious	CEA (per deaths averted)	Markov	Catch-up and follow-up measles campaigns Vs no measles campaigns
Vijayaraghavan et al. [77]	Somalia	2012	International	Maternal diseases	CEA (per LYG)	NR	Child health days strategy to deliver multiple maternal and child health interventions Vs ‘‘best buys’’ interventions
Wilcox et al. [78]	Syria	2015	National and International	CVDs	CEA (per LYG)	Decision tree	Health promotion campaign about salt reduction, labeling of salt content on packaged foods, reformulation of salt content within packaged foods, and combinations of the three strategies Vs absence of any policy
Yarahmadi et al. [79]	Iran	2010	National	Genetic disorders	CBA	NA	Newborn screening program for congenital hypothyroidism Vs no screening
Yosefy et al. [80]	Israel	2007	National	CVDs	CUA (per DALY)	NR	Nationwide program to reduce hypertension Vs no intervention
Yosefy et al. [81]	Israel	2003	National	CVDs	CUA (per QALY)	NR	Expansion of the blood pressure control program to 100 clinics nationwide Vs 30 clinics only

*3HP* 3 months of weekly rifapentine 900 mg plus isoniazid 900 mg, *6H* 6 months of daily isoniazid 300 mg, *4R* 4 months of daily rifampicin 600 mg, *HBcAb* hepatitis B core antibody, *HBsAg* hepatitis B surface antigen, *CBA* cost–benefit analysis, *CEA* cost-effectiveness analysis, *CMA* cost-minimization analysis, *CUA* cost-utility analysis, *CVDs* cardiovascular diseases, *CXR* chest X-ray, *DALYs* disability adjusted life years, *DM* diabetes mellitus, *ECG* electrocardiogram, *EGCRISC* Egyptian hepatitis C virus risk score screening tool, *FOBT* fecal occult blood test, *G6PD* glucose-6-phosphate dehydrogenase, *HCC* hepatocellular carcinoma, *HCV* hepatitis C virus, *HIV* human immunodeficiency virus, *HPV* human papillomavirus, *HPV-DNA* human papillomavirus DNA assay, *IGRA* interferon gamma release assay, *LYG* life years gained, *LYS* life years saved, *MENA* Middle East and North Africa, *NA* not applicable, *NCDs* noncommunicable diseases, *NIPT* non-invasive prenatal screening test, *NR* not reported, *NRT* nicotine replacement therapy, *Pap smear* Papanicolaou test, *QALYs* quality-adjusted life years, *QFT-Plus* QuantiFERON-TB gold plus, *TST* tuberculin skin test, VIA visual inspection with acetic acid, *Vs* versus

Geographically, this review identified 18 studies (30%) from Israel [25, 28–30, 36–40, 52, 54, 56, 58, 65, 72, 73, 80, 81], ten studies (16%) from Iran [21, 26, 41, 45, 46, 63, 67, 68, 70, 79], and five studies from Egypt [33, 34, 50, 61, 62] and Pakistan [42–44, 47, 55] (8% each). Further, the researchers obtained three studies from Afghanistan [27, 31, 76], Jordan [23, 32, 57] and Lebanon [48, 49, 71] (5% each) and two studies (3%) from Turkey [64, 69]. Only one study was identified from Algeria [24], Libya [74], Morocco [60], Oman [22], Somalia [77], Syria [78] and Tunisia [35] (12% all). Additionally, five studies (8%) were conducted in more than one MENA country [51, 53, 59, 66, 75], as shown in Table 1. In this review, 57 studies (93.4%) found public health interventions to be cost-effective or even cost-saving while only four studies (6.6%) reported non-cost-effective outcomes. The general and methodological characterestics along with the data extracted from the included studies are presented in Tables 1, 2 and 3.

**Table 2:** Methodological characteristics of the included studies

Author (references)	Year of cost estimation	Population	Perspective	Time horizon*	Discount (%)	Included costs	Model internal validity	Double counting	Sensitivity analysis
Adibi et al. [21]	2003	Premarriage individuals	Healthcare system and societal	25	3	Direct medical cost	Yes	NA	One-way and multivariate
Al Abri et al. [22]	2016 to 2017	A hypothetical cohort of 20-year-old migrants arriving in Oman	Healthcare system	Lifetime	NR	Direct medical cost	No	No	One-way, two-way and PSA
Al‐Qudah et al. [23]	2014	OPD patients with general chronic diseases at Jordan university hospital	Hospital	3 months	NA	Direct medical cost	NA	NA	One-way and multivariate
Assanelli et al. [24]	2005	Young professional and recreational athletes	NR	NR	3	Direct medical cost	No	NA	One-way
Balicer et al. [25]	2018	Whole population	Healthcare system and societal	NR	NR	Direct and indirect costs	NA	NA	Multivariate
Barfar et al. [26]	2019	Low socioeconomic women aged 35 and higher	Healthcare system	1	NA	Direct medical cost	No	NA	One-way
Carvalho et al. [27]	2014	Women aged 15–45	NR	Lifetime	NR	Direct and indirect costs	Yes	NA	One-way and PSA
Chodick et al. [28]	2015	Physician and nurses aged < 45 years	Employer (healthcare payer)	20	3	Direct medical cost	No	NA	One-way
Chodick et al. [29]	2016	Healthcare workers	Healthcare system	20	3	Direct costs	No	No	One-way
Chowers [30]	2014	All pregnant women	Payer	100	3.5	Direct medical cost	Yes	No	Univariate and multivariate
Devine [31]	2017	Adult patients with vivax malaria	Healthcare provider	1	NA	Direct costs	No	No	One-way and PSA
El-Dahiyat [32]	2016	The whole population	NR	NR	NR	Product price	NA	NA	NR
El-Ghitany [33]	2012	People at intermediate and high risk scores for HCV infection	NR	NR	NR	Cost of testing	No	NA	NR
Eltabbakh et al. [34]	2015	Cirrhotic patients older than 18 years	NR	NR	NR	Direct medical cost	No	No	NR
Gamaoun et al. [35]	2010	Young adolescent girls (12 years) and women 35–59 years	NR	NR	3	Direct medical cost	No	NA	NR
Ginsberg et al. [36]	2011	Pregnant women, couples before marriage, relatives of subjects with thalassemia, and even school children	Healthcare system and societal	30	5	Direct and indirect costs	NA	NA	One-way
Ginsberg et al. [37]	2014	Females aged 12–65 years	Healthcare system	Lifetime	3	Direct costs	No	No	One-way
Ginsberg et al. [38]	2018	Men who have sex with men	Societal	NR	3	Direct costs	No	No	One-way
Ginsberg et al. [39]	2016	Females aged 12–65 years old	Healthcare system	100	3	Direct costs	No	No	One-way
Ginsberg et al. [40]	2019	Adults aged 20 and above	NR	1	NA	Direct and indirect costs	Yes	Yes	One-way
Haghighat et al. [41]	2017	Women aged 40–70 years	Healthcare system	50	3 and 5	Direct costs	No	No	One-way and PSA
Hamdani et al. [42]	2013	Primary care attendees with high levels of psychological distress and functional impairment	Healthcare system	1	NA	Direct costs	Yes	NA	NR
Howard et al. [43]	2017	Afghan refugee resided in Pakistan	Societal	5	3	Direct and indirect costs	No	Yes	Univariate
Hussain et al. [44]	2018	TB patients who had been on treatment for a minimum of 2 months	Patients, health facility and TB program	2	3	Direct and indirect costs	No	No	One-way
Javadinasab et al. [45]	2016	First-degree relatives (aged 40 years and above) of patients with colorectal cancer	Healthcare system	Lifetime	5	Direct medical cost	Yes	No	One-way and deterministic
Javanbakht [46]	2014	Adults and the elderly population	Healthcare system	20	NR	Direct costs	Yes	NA	PSA
Kashi et al. [47]	2013	Pregnant women	NR	Lifetime	3	Direct costs	No	No	PSA
Khneisser et al. [48]	2010	All newborn babies	NR	NR	NR	Direct costs	NA	NA	NR
Khneisser et al. [49]	2014	All male newborns	NR	NR	NR	Direct medical cost	NA	NA	NR
Kim et al. [50]	2013	40-year-old and asymptomatic- HCV average-risk adults in Egypt	Societal	40	3	Direct and indirect costs	Yes	Yes	One-way, two-way and PSA
Kim et al. [51]	2013	Pre-adolescent girls (by age 9)	Societal	Lifetime	3	Direct and indirect costs	Yes	Yes	One-way
Koren et al. [52]	2017	Pregnant women, husbands of affected women, and patients with β Thalassemia major	NR	50	NR	Direct medical cost	No	NA	NR
Lahiri et al. [53]	2104	The entire economically active population	Societal	100	3	Direct costs	No	NA	One-way
Leshno et al. [54]	2014	Average risk population (50 years and over)	NR	Lifetime	3	Direct costs	No	NA	One-way and two-way
Lim et al. [55]	NR	General population and people who inject drugs	Healthcare provider	NR	3.5	Direct medical cost	Yes	NA	One-way
Lohse et al. [56]	NR	Pregnant women	NR	NR	3	Direct costs	Yes	No	NR
Madae’en et al. [57]	2012	Hypothetical cohort of Jordanian male smokers aged 30 years or older	Public payer (MoH)	70	3	Direct medical costs	No	NA	One-way and PSA
Marseille et al. [58]	2014	Pregnant women	NR	Lifetime	3	Direct costs	No	No	One-way and multivariate
Mason et al. [59]	2016	General population	NR	10	3	Direct costs	No	NA	One-way
Messoudi et al. [60]	2008	Girls at 14 years and Women aged 30–49	Healthcare system	Lifetime	3	Direct medical cost	Yes	NA	One-way and two-way
Mostafa et al. [61]	2018	Population exposed to unsafe injection practices	Healthcare system	30	3.5	Direct medical cost	Yes	No	One-way
Mostafa et al. [62]	2008	Population exposed to unsafe injection practices (aged 15–59 years)	Healthcare system	26	3.5	Direct medical cost	Yes	No	One-way
Nahvijou et al. [63]	2012	Women over 15 years of age	Healthcare system	Lifetime	3	Direct medical costs	Yes	No	One-way
Okem et al. [64]	2013	Pregnant women	Payer	NR	NR	Direct medical cost	No	NA	NR
Ornoy et al. [65]	NR	People with attention deficit hyperactivity disorder	Societal	Lifetime	NR	Direct and indirect costs	NA	NA	NR
Ranson et al. [66]	2016	Cohort of smokers	Public sector provider	30–50	3–10	Direct and indirect costs	No	No	NR
Rashidian et al. [67]	2017	Iranian hospitals	Provider	10	5–10	Direct and indirect costs	No	NA	One-way
Rezaei-Hemami et al. [68]	2017	NR	MoH	1	NA	Direct costs	No	NA	One-way
Saygili et al. [69]	2012	Cancer patients receiving palliative care	Societal and patient	1 Month	NA	Direct and indirect costs	No	NA	One-way
Shamshiri et al. [70]	2016	3 – 5 days old neonates	Caregiver	82	3	Direct costs	Yes	No	One-way
Sharma et al. [71]	2012	Women aged 25–65 years	Societal	Lifetime	3	Direct costs	No	NA	One-way
Shlomai et al. [72]	2011	The whole population	MoH	200 Days	NA	Direct and work absence costs	Yes	Yes	One-way and PSA
Shmueli et al. [73]	2015	Moderate-to-heavy smokers aged 45 years or older	Healthcare system	Lifetime	3	Direct medical cost	Yes	No	One-way and PSA
Sladkevicius et al. [74]	2016	Neonates	Societal	Lifetime	3	Direct and indirect costs	No	NA	One-way and PSA
Verguet et al. [75]	2019	Heterosexual adult population (15–49-year-old)	NR	5	NR	Direct medical cost	No	No	One-way
Vijayaraghavan et al. [76]	2008	12 million children aged six months to 12 years and 5 million children aged 9 to 59 months	Donor	10	3	Direct costs	Yes	NA	One-way
Vijayaraghavan et al. [77]	2010	Children and women of childbearing age in populations not reached by routine health services in a conflict setting	Donor	2	NR	Direct medical costs	No	NA	One-way
Wilcox et al. [78]	2013	Whole population	Healthcare system	10	3	Direct costs	Yes	NA	Multi-way
Yarahmadi et al. [79]	2010	Newborn babies	NR	70	3	Direct medical costs	NA	NA	NR
Yosefy et al. [80]	2018	Adults aged 25–64 years	NR	20	3	Direct costs	No	No	One-way
Yosefy et al. [81]	2017	All hypertensive patients	NR	10	4	Direct costs	No	No	One-way

*OPD* out-patient department, *HCV* hepatitis C virus, *PSA* probabilistic sensitivity analysis, *MoH* Ministry of Health, *NA* not applicable, *NR* not reported, *TB* tuberculosis

^*^In years unless otherwise stated

**Table 3:** Sources of input parameters and results

Author (references)	Source of cost data	Source of epidemiological data	Source of effectiveness data	Source of utility data	Source of funding	Threshold	ICER and results	Conclusion
Adibi et al. [21]	Iranian	Iranian, international and expert consensus	International	NA	Academia	1 GDP (US$ 1790 in 2003)	The cost/CHB infection averted was US$ 202 and 197 for the strategies 1 and 2, respectively	Premarriage prevention of HBV transmission in Iran seems cost saving
Al Abri et al. [22]	Omani and international	Omani and international	International	International	Industry	WTP of US$ 100,000 in 2020	The QFT-Plus with 3HP was more cost-effective than the other TB strategies with an ICER of US$ 2915/QALY gained. The CXR strategy was the least cost-effective	IGRA testing followed by 3HP is the most cost-effective intervention
Al‐Qudah et al. [23]	Jordanian and assumption	Jordanian	International	NA	None	NA	Benefit‐to‐cost ratio was 5.98 and an annual net benefit was US$ 64,393	Clinical pharmacist intervention is cost beneficial and offers substantial cost savings to the healthcare payer
Assanelli et al. [24]	Algerian	International	International	NA	Industry	NR	The total cost in Algeria was $PPP 79,395, total cost/athlete was $PPP 74.10, and CER of screening was $PPP 582	Results strongly support the utilization of 12-lead ECG in the pre participation screening of young athletes
Balicer et al. [25]	Local	International	International	NA	NR	NA	Therapeutic treatment and postexposure prophylaxis were shown to be cost-saving, with a cost–benefit ratio of 2.44–3.68	Pre pandemic stockpiling of Oseltamivir is cost-saving to the economy and to the healthcare system, if the use is limited to treat patients at high risk
Barfar et al. [26]	Iranian	Iranian	International	NA	Government	NR	ICER/breast cancer detected was US$ 15,742	Mammography screening program is not cost-effective
Carvalho et al. [27]	WHO CHOICE, donors and local	Afghan and international	NR	NA	NGO	1–3 GDP/C (US$ 500–1500 in 2009/10)	ICERs of family planning strategies were below US$ 130/LYG. ICERs of stepwise improvements in maternal health services were below US$ 200/LYG	The combination of investment in reproductive health infrastructure and increase in family planning is highly cost-effective
Chodick et al. [28]	Local and international	Local	International	NA	NR	NR	The incremental cost of screening and vaccination of susceptible workers was US$ 23,713/avoided case, serological tests was US$ 206,692/avoided case, and vaccinating all HCWs without serotesting wad US$ 10.4 million/avoided case	Screening and vaccination of susceptible workers using anamnestic selection are cost-effective while screening alone and mass vaccination alone of all HCWs without serotesting are not cost-effective
Chodick et al. [29]	Local and international	Local and international	International	International	Government	US$ 60,000	Screening prior vaccination among 18- to 39-year-old physicians and paramedical workers achieved the lowest cost per prevented Hepatitis A case (US$ 6240 and 6773, respectively). ICERs/QALY were US$ 56,532 and 61,350 for the same groups	Screening followed by selective vaccination for physicians and for paramedical workers is recommended
Chowers [30]	Local	Local and international	International	Local and international	NR	1–3 GDP/C (US$ 28,667–86,000) in 2014)	Universal prenatal screening dominates over the current policy with an ICER of (US$ -11,546)/QALY gained	Universal prenatal HIV screening is projected to be cost saving
Devine [31)	International	International	International	International	Government, academia and NGO	1 GDP/C	The ICERs were US$ 18.6 for 14-day Primaquine (without G6PD screening), US$ 1089 for Tafenoquine in male and 7-day Primaquine in female (both with G6PD screening)	Using a gender-based treatment strategy could significantly change the landscape for providing the radical cure of *Plasmodium vivax Malaria*
El-Dahiyat [32]	Jordanian	NA	NA	NA	None	NA	The average savings if using the generic drugs instead of the originator brand medicines in Jordan was 32.68%, and the maximum savings was 74.29%	Generic substitution can provide significant savings to patients and healthcare system
El-Ghitany [33]	Egyptian	Egyptian	NR	NA	None	NR	Using EGCRISC would save LE 0.43 billion accounting for about 21,646,227 unnecessary tests	EGCRISC is a cost-effective tool that must be adopted nationwide
Eltabbakh et al. [34]	Egyptian	NR	NR	International	NR	1–3 GDP/C (US$ 3184–9553)	ICER was not reported. The costs were US$ 1105 and 1180/QALY for screening with ultrasound only and for both ultrasound and alpha-fetoprotein, respectively	Screening for HCC is highly cost-effective
Gamaoun et al. [35]	Tunisian, international and estimations	Tunisian and international	Tunisian and international	NA	NR	NR	The incremental cost of cervical cancer screening according to 10-year periodicity was US$ 8219, 5-year periodicity was US$ 14,567, 3-year periodicity was US$ 20,479, and finally the national vaccination program was US$ 36,854 per avoided cervical cancer case	Cervical cancer screening each 5 years combined with scheduled two-dose anti-HPV national vaccination program is the best cost-effective strategy for cervical cancer prevention
Ginsberg et al. [36]	Local and assumptions	Local	Local and international	NA	NR	NA	The benefit–cost ratio of the program to the health services was 4.22:1 which increased to 6.01:1 when a societal perspective was taken	The monetary benefits of a nationwide thalassemia screening program to society and to the healthcare system exceeds the program's costs
Ginsberg et al. [37]	Local	Local and international	International	International	NR	1–3 GNP/C (US$ 27,055–81,165 in 2010)	ICER/DALY averted was US$ 2509 for Pap smear screening of females at age 40, US$ 10,543 for thrice a lifetime VIA, US$ 22,841 for three doses HPV vaccination at age 12 plus a booster dose at ages 32 and 52 combined with penta-annual Pap smear screening for females aged 20–65, and US$ 30,029 for addition of penta-annual HPV DNA screening to vaccination and penta-annual Pap smear	HPV screening interventions combined with vaccination program have the potential to be very cost-effective
Ginsberg et al. [38]	Local	Local	International	International	None	1–3 GDP/C (US$ 40,439–121,316 in 2017)	ICER of HIV pre-exposure prophylaxis drugs was around US$ 967,744/averted DALY	HIV pre-exposure prophylaxis drugs were found not to be cost-effective. Prices would have to fall by 90.7% for the intervention to become cost-effective
Ginsberg et al. [39]	Local and international	Local	International	NR	NR	1–3 GDP/C (US$ 20,366–61,098 in 2007)	ICER/QALY gained were US$ 65,024 for annual Pap smear, US$ 35,403 for tri-annual Pap smears, US$ 28,612 for penta-annual Pap smears, US$ 9,273 for thrice a lifetime Pap smears, US$ 48,660 for tri-annual Pap smears with HPV-DNA testing, US$ 33,705 for penta-annual combination, US$ 46,807 for a thrice a lifetime HPV-DNA testing, US$ 61,264 for thrice a lifetime VIA, US$ 81,404 for one-off HPV vaccination females aged 12, US$ 272,010 for vaccinating females every 10 years from age 12 to 62	All HPV screening interventions are cost effective or highly cost-effective except for annual Pap smear and a thrice a lifetime VIA. HPV vaccination program is not cost-effective as well
Ginsberg et al. [40]	Local and international	Local	NA	NR	NR	1–3 GNP/C (NIS 104,161–312,483 in 2010)	Implementation of the cluster of interventions would save 32,671 QALYs at a cost of NIS 47,559/QALY	Fielding an eight-pronged combined clinical and community-based dietary interventional program is very cost-effective
Haghighat et al. [41]	Iranian	Iranian and international	Iranian	International	None	1–3 GDP/C (Int. $ 13,100–39,300 in 2012)	ICERs of mammography screening were Int. $ 37,350, Int. $ 141,641 and Int. $ 389,148/QALY gained in the first, second and third rounds of screening program, respectively	Mammography screening program is cost effective in 53% of the cases, but ICER/QALY in the second and third rounds of screening are not cost-effective
Hamdani et al. [42]	Pakistani	Pakistani	Pakistani	NA	Government	US$ 67	The mean ICER to successfully treat a case of depression using an international supervisor was US$ 517 compared with US$ 102.93 using a local one	The Problem Management + is more effective but also more costly
Howard et al. [43]	Pakistani	Pakistani and international	NR	Afghan	None	1–3 GDP/C (US$ 479–1436 in 2015)	The additional cost of including indoor residual spraying over five years per case prevented was US$ 39 (50 for *Vivax* and 182 for *Falciparum*). Per DALY averted this was US$ 266	Adding indoor residual spraying is cost-effective
Hussain et al. [44]	Pakistani	NR	International	International	NGO	NR	Incentive-based active case finding program costs US$ 223 per patient treated and incrementally averted 0.17 DALYs at the cost of US$ 15.74 over 6 months	Both screening strategies appear to be cost-effective in an urban Pakistani context
Javadinasab et al. [45]	Iranian	Iranian and international	Iranian and international	International	NR	1–3 GDP/C (US$ 5442–16,326 in 2014)	In CUA, compared with no screening, the ICERs/QALY gained were US$ 489 for one screening/lifetime at age 50, US$ 709 for one screening/lifetime at age 55, US$ 1010 for screening every 10 years starting at age 50, US$ 1386 for screening every 10 years starting at age 40, US$ 2310 for screening every 5 years starting at age 50 and US$ 3135 for screening every 5 years starting at the age of 40. In CEA, compared with no screening, the ICERs/LYG gained were US$ 725 for one screening/lifetime at age 50, US$ 1115 for one screening/lifetime at age 55, US$ 1540 for screening every 10 years starting at age 50, US$ 1995 for screening every 10 years starting at age 40, US$ 3508 for screening every 5 years starting at age 50, and US$ 4489 for screening every 5 years starting at the age of 40	Colorectal cancer colonoscopy screening in high-risk individuals is cost-effective in Iran. Colonoscopy screening every 10 years starting at the age of 40 is the most cost-effective strategy
Javanbakht [46]	Iranian	Iranian and international	NA	NA	Academia	NR	The estimated savings in health cost per capita were US$ 0.43, 8.42, 39.97 and 190.25 in 1, 5, 10 and 20-years’ time horizons, respectively. Corresponding total aggregated avoidable costs for entire population were US$ 33.83 million, 661.31 million, 3138.21 million and 14,934.63 million, respectively	Increasing dairy foods consumption to recommended levels would be associated with reductions in healthcare costs
Kashi et al. [47]	Estimation	International	International	International	NGO	1–3 GDP/C	The ICER of transitioning from iron and folic acid supplementation to multiple micronutrient supplementation was US$ 41.54/DALY in Pakistan	Multiple micronutrient supplementation is cost-effective and generates positive health outcomes for both infants and pregnant women
Khneisser et al. [48]	Lebanese	Lebanese	NR	NA	Academia	NA	A reduction by half of direct cost of care, reaching on average US$ 31,631 per detected case was shown. This difference more than covers the expense of starting a newborn screening program	Direct and indirect costs saved through early detection of these disorders are important enough to justify universal publicly-funded screening, especially in developing countries with high consanguinity rates
Khneisser et al. [49]	Lebanese	Lebanese	NR	NA	Academia	NA	The cost–benefit index of systematic screening was about 2.58 times lower than that of anemia-related hospitalizations in an unscreened population	The efficiency of routinely testing described in this study supports changes in screening policies for boys
Kim et al. [50]	Egyptian	Egyptian and international	Egyptian and international	International	Academia	1–3 GDP/C (US$ 3333–10,000 in 2014)	No screening would cost US$ 1840 for 19.179 QALYs. Implementing a screening program using triple-therapy was dominant compared to no screening because it would have lower total costs (US$ 1816) and lead to higher QALYs (19.229)	Screening and treatment programs for HCV in Egypt can be cost-effective methods to reduce the burden of liver disease
Kim et al. [51]	Local, regional and assumption	Local and international	Local and international	International	NGO and public	1 GDP/C (Int. $ 7521 in Algeria, 12,605 in Lebanon and 12,540 in Turkey; all in 2010 values)	Cytology-based screening alone was less cost effective, in Lebanon, Turkey and Algeria. The CER for combined vaccination and cytology screening was Int. $ 7520 in Algeria and 12,540 in Turkey while it was not cost-effective in Lebanon	Annual cytology screening is not cost-effective. Promoting organized, less frequent (3–5 years) screening and adopting HPV DNA testing can result in more efficient cervical cancer prevention efforts
Koren et al. [52]	Local	Local	NR	NA	Industry	NR	The cost of preventing one affected newborn was US$ 63,660 compared to 1,971,380 for treatment of a patient during 50 years	Implementation of a national β Thalassemia prevention program appears cost-effective
Lahiri et al. [53]	Local, regional and assumptions	International	International	NA	NGO	NR	In all of the sub-regions, training was the most cost-effective with CER of US$ 74 per LYG in the sub-region comprising of Egypt, Iraq, Morocco and Yemen so it would be the first choice option where resources are scarce	Worker training is a low cost and feasible first step toward reducing back pain/injury incidence. However, the engineering controls interventions as well as the full ergonomics program look very cost effective for all of the WHO sub-regions
Leshno et al. [54]	Local	Local, international and estimations	International	NA	Industry	NR	Annual FOBT plus sigmoidoscopy during a 5-year interval was the best strategy with an ICER of NIS 1268/LYG	It is highly cost-effective to screen average-risk asymptomatic individuals beginning at age 50. One-time colonoscopic screening or FOBT plus sigmoidoscopy would be the preferred options
Lim et al. [55]	Pakistani, international and assumption	Pakistani and international	NR	NA	NGO	NR	Screening and treatment strategy will cost US$ 3.9 billion over 13 years with the yearly costs making up 9% of the annual health budget of Pakistan. This translates to about US$ 600/cure	Pakistan needs to invest up to 9% of its yearly health expenditure (0.11% of its GDP, or approximately US$ 1.50 /person/year) to achieve the WHO HCV-elimination target
Lohse et al. [56]	Local	International	International	NR	Industry	1 GDP/C (US$ 29,500 in 2010)	The full costs of universal screening of pregnant women was US$ 5887/DALY	GDM screening and postpartum lifestyle management have an attractive cost-effectiveness ratio
Madae’en et al. [57]	Jordanian	Jordanian	International	NA	None	1–3 GDP/C (US$ 4395–13,185 in 2019)	103,970 and 64,030 life years were gained using the Varenicline and NRT regimen compared to the no-intervention arm. The costs per LYG were US$ 1696 and US$ 1890 for Varenicline and NRT, respectively	Provision of Varenicline is a cost-effective intervention. Also, provision of NRT is likely to be cost-effective
Marseille et al. [58]	Local, international and assumptions	Local, international and estimations	International	International	Industry	1–3 GDP/C (US$ 29,800–89,400 in 2010)	The program cost/1000 pregnant women was US$ 259,929. The cost/DALY averted was US$ 1830	By WHO standards, GDM interventions are highly cost-effective
Mason et al. [59]	Local (from each country)	NR	International	NA	Academia and NGOs	NR	In all four countries most policies were cost saving compared with the baseline. The combination of all three policies resulted in estimated cost savings of US$ 235,000,000 and 6455 LYG in Tunisia; US$ 39,000,000 and 31,674 LYG in Syria; US$ 6,000,000 and 2682 LYG in Palestine and US$ 1,3000,000,000 and 378,439 LYG in Turkey	Reducing dietary salt intake will reduce CHD deaths in the four countries. Having a comprehensive health education strategy and food industry procedures for labeling and minimizing salt content would save money and lives
Messoudi et al. [60]	Moroccan, regional and international	Moroccan	International	NA	Government, academia, NGO and industry	1–3 GDP/C (US$ 2860–8580 in 2018)	The costs were US$ 551/LYS for current VIA screening and US$ 1150/LYS for HPV vaccination of pre-adolescent girls compared to no intervention. The cost of combined strategy of HPV vaccination and current screening was US$ 2843/LYS compared to screening alone	Current screening would be good value for money compared with no intervention but would be inefficient compared with vaccination
Mostafa et al. [61]	Egyptian	Egyptian	International	International	NGO	NR	Using Safety-engineered syringes was dominant option (less costly and more effective) with an ICER of US$ − 1802/QALY gained compared to conventional syringes	Using Safety-engineered syringes is more effective and cost-saving strategy
Mostafa et al. [62]	Egyptian	Egyptian and international	NR	International	NGO	NR	Using Safety-engineered syringes was dominant option (less costly and more effective) with an ICER of Int. $ − 18,650/QALY gained compared to conventional syringes	Using Safety-engineered syringes is cost saving prevention policy
Nahvijou et al. [63]	Iranian and assumptions	Iranian and international	Iranian and international	International	Academia	1–2 GDP/C (US$ 6631–13,262 in 2013)	Compared with no-screening strategy, the most cost-effective strategy (ICER of US$ 8875/QALY) was HPV DNA testing beginning at age 35 years with 10-year screening intervals	Organized cervical screening with HPV DNA testing for women is recommended, beginning at age 35 and repeated every 10 or 5 years
Okem et al. [64]	Turkish	Turkish	International	NA	NR	NR	ICER of NIPT was PPP 17,235,174/Down syndrome cases detected compared to combined test. ICER of NIPT following combined test was PPP 6,873,082/Down syndrome cases detected compared to combined test	NIPT leads to very high costs despite its high effectiveness. Thus, cost of NIPT should be decreased
Ornoy et al. [65]	Local and assumptions	Local	NA	NA	None	NA	The benefit cost ratio was 7.02 and, assuming only 50% success of treatment, it was 3.51	National screening program offers a very high cost benefit ratio
Ranson et al. [66]	Local, regional and international	Local, regional and international	Local, regional and international	NR	NGO	NR	Tax increases to raise the real price of cigarettes by 10% worldwide would prevent between 5 and 16 million tobacco-related deaths, and could cost US$ 3–70/DALY saved in LMIC. NRT and a package of non-price interventions other than NRT were also cost-effective in LMIC, at US$ 280–870 and US$ 36–710/DALY, respectively. In HIC, price increases were found to have a cost-effectiveness of US$ 83–2771/DALY, NRT US$ 750–7206/DALY and other non-price interventions US$ 696–13,924/DALY	Tobacco control policies, particularly tax increases on cigarettes, are cost-effective relative to other health interventions
Rashidian et al. [67]	Iranian	International	National	NA	Government	NR	Caspian-Alborz device was the most cost-effective alternative with an average cost-effectiveness from US$ 33 to 333/treatment of every one cubic meter of infectious waste in various conditions	There is more than one cost-effective device for different conditions and times in a country
Rezaei-Hemami et al. [68]	Iranian	Iranian and international	Iranian and international	NA	Academia	NR	The most cost-effective interventions were the use of insecticide-treated nets, Larviciding, surveillance for diagnosis and treatment of patients in less than 24 h, and indoor residual spraying, respectively	Insecticide-treated net is the most cost effective intervention
Saygili et al. [69]	Turkish	Local and international	NR	NA	NR	NR	From a societal perspective, palliative care services provided at hospital IPD were more cost‐effective. From a patient perspective, home healthcare was more cost‐effective with an ICER of US$ 33.43 and US$ -18.30/QALY compared to hospital IPD and comprehensive palliative care center, respectively	Hospital inpatient palliative care is more cost‐effective compared with other alternatives from societal perspective
Shamshiri et al. [70]	Iranian	Iranian	NR	Iranian	Academia	NR	ICERs for screening programs with different TSH cut-off points versus no screening were similar (US$ − 4.5 ± 0.2/DALY)	The current threshold of TSH in the national congenital hypothyroidism screening program is the most cost-effective threshold
Sharma et al. [71]	Lebanese and international	Lebanese	International	NA	None	1 GDP/C (Int. $ 17,462 in 2014)	ICERs/LYG were Int. $ 80,670 for annual cytologic screening at 20% coverage, Int. $ 12,210 for HPV DNA testing screening every 5 years at 50% coverage and Int. $ 16,340 for HPV DNA testing every 4 years at 50% coverage	Screening each 5 and 4 years is cost effective but annually is not
Shlomai et al. [72]	NR	Local	NA	NR	None	WTP of US$ 50,000–150,000	The ICER would be US$ 45.1 million/one death case prevented and US$ 15.24 million/QALY	A national lockdown strategy has a moderate advantage in saving lives with extremely high costs and possible overwhelming economic effects
Shmueli et al. [73]	Local	Local	International	Local	NGO and academia	WTP of US$ 10,000 and 20,000	ICER/QALY gained by screening was US$ 1464	Screening presents a good value for the money and should be considered for inclusion in the national list of health services financed publicly
Sladkevicius et al. [74]	Libyan	National, regional and international	International	NA	Industry	WTP of US$ 4,000	The expected cost/undiscounted LYG was US$ − 15,500. There would be a 90% return on investment in the screening program since society would gain US$ 1.9 for every invested US$ 1	Screening program is cost-effective from a societal perspective
Verguet et al. [75]	Regional and international	International	International	International	NR	1–3 GDP/C	ICERs/DALY were US$ 12,300 in Djibouti, 41,000 in Mauritania, 41,600 in Somalia and 19,600 in Sudan	Adding HIV pre-exposure prophylaxis is not cost-effective in Djibouti, Mauritania, Somalia, and Sudan due to low levels of HIV burden and high levels of male circumcision
Vijayaraghavan et al. [76]	National and international	National and international	International	NA	NR	1–3 GNI/C (US$ 735–2205 in 2002)	The cost/death prevented was US$ 23.6. For every one million US$ invested by donors, an estimated 42,300 deaths were prevented by the campaigns. For the same investment, the catch-up campaign averted 43,700 deaths while the follow-up campaign averted 38,300 deaths	The campaigns were extremely cost-effective and provided excellent returns on investment under all scenarios considered in the analysis
Vijayaraghavan et al. [77]	Somali	International	International	NA	Donors	1 GNI/C (US$ 140 in 2010)	The cost-effectiveness ratios were US$ 44/LYS by 1st round, US$ 28/LYS by 2nd round and US$ 34/LYS by both rounds combined. For every US$ 1 million invested in both rounds, an estimated 615 children’s lives, or 29,500 life years, were saved	Child Health Days are very cost-effective strategy for addressing the leading causes of children mortality in a conflict setting like Somalia
Wilcox et al. [78]	Syrian	Syrian	International	NA	Academia	PPP$ 13,000–38,997	CERs/LYG were PPP$ 5453 for reformulation of salt content within packaged foods, PPP$ 2201 for combination of health promotion campaign and reformulation of salt content and PPP$ 2125 for combination of reformulation of salt content and labeling of salt content on packaged foods	All salt reduction policies are cost-saving or cost effective
Yarahmadi et al. [79]	NR	Local	International	NA	NR	NR	Benefit to cost ratios with regard to education and care of patients with mental retardation were lower by 22 times (100% in the public sector), 41 times (100% in the private sector), 32 times (50% in the public sector and 50% in the private sector), 34 times (100% in the public sector day and night), 47 times (50% in the public sector and 50% in the private sector day and night), and 60 times (100% in the private sector day and night)	Newborn screening program for congenital hypothyroidism has been quite effective
Yosefy et al. [80]	Local	Local and International	NR	Local	NR	1–3 GDP/C (US$ 16,497–49,491 in 2003)	The implementation of health education program nationwide was likely to save over 2000 lives and US$ 185 million in health care resources alone	It is conceivable that the health education program may be extended not only throughout this country, but also to neighboring countries
Yosefy et al. [81]	Local	NR	Local	International	Government	NR	The net saving to health services would be US$ 977,993 and the increase in QALYs would be 602 years	Better control of hypertensive patients is cost effective

*3HP* 3 months of weekly rifapentine 900 mg plus isoniazid 900 mg, *HBcAb* hepatitis B core antibody, *HBsAg* hepatitis B surface antigen, *CBA* cost–benefit analysis, *CEA* cost-effectiveness analysis, *CER* cost-effectiveness ratio, *CHB* chronic hepatitis B, *CHD* coronary heart disease, *CMA* cost-minimization analysis, *CUA* cost-utility analysis, *CVDs* cardiovascular diseases, *CXR* chest X-ray, *DALYs* disability adjusted life years, *DM* diabetes mellitus, *ECG* electrocardiogram, *EGCRISC* Egyptian hepatitis C virus risk score screening tool, *FOBT* fecal occult blood test, *G6PD* glucose-6-phosphate dehydrogenase, *GDM* gestational diabetes mellitus, *GDP/C* gross domestic product per capita, *GNI/C* gross national income per capita, *GNP/C* gross national product per capita, *HBV* hepatitis B virus, *HCC* hepatocellular carcinoma, *HCV* hepatitis C virus, *HCWs* healthcare workers, *HIC* high income countries, *HIV* human immunodeficiency virus, *HPV* human papillomavirus, *HPV-DNA* human papillomavirus DNA assay, *ICER* incremental cost-effectiveness ratio, *IGRA* interferon gamma release assay, *Int. $* international dollar, *IPD* inpatient department, *LE* Egyptian pound, *LMIC* low and middle income countries, *LYG* life years gained, *LYS* life years saved, *NA* not applicable, *NCDs* noncommunicable diseases, *NGO* non-governmental organization (non-for-profit), *NIPT* non-invasive prenatal testing, *NIS* New Israeli Shekels, *NR* not reported, *NRT* nicotine replacement therapy, *Pap-smear* Papanicolaou test, *QALYs* quality-adjusted life years, *QFT-Plus* QuantiFERON-TB gold plus, *PPP* purchasing power parity, *SR* Saudi Riyal, *TB* tuberculosis, *TSH* thyroid stimulating hormone, *TST* tuberculin skin test, VIA visual inspection with acetic acid, *WHO* World Health Organization, *WHO-CHOICE* World Health Organization-CHOosing Interventions that are Cost-Effective, *WTP* willingness-to-pay

### Quality assessment

Table 4 presents the quality assessment results using the CHEERS checklist. Overall, study quality was good, with a median of 82.6% of applicable items being met (SD 14%). Forty-four studies (72%) achieved scores above an arbitrary threshold of 75%; therefore, they were considered high quality and potentially useful in policy decision-making [18, 19, 82]. The mean (SD) of items reported was 80.8% (14%). The evaluation of reporting adequacy guided by the CHEERS checklist resulted in scores ranging from 8 (35%) to 24 (100%), as shown in Table 4. Of the 61 studies included in this review, five studies (8%) [21, 41, 44, 51, 60] were deemed to be of excellent quality and 39 studies (64%) [22–24, 26–31, 36–40, 42, 43, 45–47, 50, 53–55, 57–59, 61–63, 66, 67, 70–74, 76–78] of high quality. However, 16 studies (26%) [25, 32, 34, 35, 48, 49, 52, 56, 64, 65, 68, 69, 75, 79–81] and one study (2%) [33] were incomplete with respect to important methodological details and were considered to be of average and poor quality, respectively*.*

**Table 4:** Study quality assessment by CHEERS checklist

Author (References)	Title	Abstract	Introduction	Population	Setting/location	Perspective	Comparators	Time horizon	Discount rate	Outcome measures	Effectiveness	Preference based Outcomes
Adibi et al. [21]	+	+	+	+	+	+	+	+	+	+	+	NA
Al Abri et al. [22]	+	−	+	+	+	+	+	+	−	+	+	+
Al‐Qudah et al. [23]	+	+	+	+	+	+	+	+	NA	+	−	NA
Assanelli et al. [24]	+	+	+	+	+	−	−	−	+	+	+	NA
Balicer et al. [25]	+	−	+	+	+	+	+	−	−	+	+	NA
Barfar et al. [26]	+	+	+	+	+	+	+	+	+	+	+	NA
Carvalho et al. [27]	+	+	+	+	+	−	+	+	−	+	−	NA
Chodick et al. [28]	+	+	+	+	+	+	+	+	+	+	+	NA
Chodick et al. [29]	+	+	+	+	+	+	+	+	+	−	+	+
Chowers [30]	+	+	+	+	+	+	+	+	+	−	+	−
Devine [31]	+	+	+	+	+	+	+	+	NA	+	+	+
El-Dahiyat [32]	+	+	+	+	+	−	+	−	−	NA	NA	NA
El-Ghitany [33]	+	+	+	+	−	−	+	−	−	−	−	NA
Eltabbakh et al. [34]	+	+	+	+	+	−	+	−	−	+	−	+
Gamaoun et al. [35]	+	+	+	+	+	−	+	−	+	+	+	NA
Ginsberg et al. [36]	+	+	+	+	+	+	+	+	+	+	−	NA
Ginsberg et al. [37]	+	−	+	+	+	+	+	−	+	+	−	−
Ginsberg et al. [38]	+	+	+	+	+	+	+	−	+	−	+	+
Ginsberg et al. [39]	+	−	+	+	+	+	+	+	+	+	+	+
Ginsberg et al. [40]	+	+	+	+	+	−	+	+	NA	+	NA	+
Haghighat et al. [41]	+	+	+	+	+	+	+	+	+	+	+	+
Hamdani et al. [42]	+	+	+	+	+	+	+	+	+	+	+	NA
Howard et al. [43]	+	+	+	+	+	+	+	+	+	+	−	+
Hussain et al. [44]	+	+	+	+	+	+	+	+	+	+	+	+
Javadinasab et al. [45]	+	+	+	+	+	+	+	+	+	+	+	−
Javanbakht [46]	+	+	+	+	+	+	−	+	−	+	NA	NA
Kashi et al. [47]	+	+	+	+	+	−	+	+	+	+	+	+
Khneisser et al. [48]	+	−	+	+	+	−	+	−	−	−	−	NA
Khneisser et al. [49]	+	−	+	+	+	−	+	−	−	+	−	NA
Kim et al. [50]	+	+	+	+	+	+	+	+	+	+	+	+
Kim et al. [51]	+	+	+	+	+	+	+	+	+	+	+	NA
Koren et al. [52]	+	+	+	+	+	−	+	+	−	−	−	NA
Lahiri et al. [53]	+	+	+	+	+	+	+	+	+	+	NA	NA
Leshno et al. [54]	+	−	+	+	+	−	+	+	+	+	+	NA
Lim et al. [55]	+	+	+	+	+	+	+	−	+	+	−	NA
Lohse et al. [56]	+	+	+	−	+	−	+	−	+	+	+	−
Madae’en et al. [57]	+	+	+	+	+	+	+	+	+	+	+	NA
Marseille et al. [58]	+	+	+	−	+	−	+	−	+	+	+	+
Mason et al. [59]	+	+	+	+	+	−	+	+	+	+	+	NA
Messoudi et al. [60]	+	+	+	+	+	+	+	+	+	+	+	NA
Mostafa et al. [61]	+	+	+	+	+	+	+	+	+	+	+	+
Mostafa et al. [62]	+	+	+	+	+	+	+	+	+	+	+	+
Nahvijou et al. [63]	+	+	+	+	+	+	+	+	+	+	+	+
Okem et al. [64]	+	+	+	+	+	+	+	−	−	+	+	NA
Ornoy et al. [65]	+	+	+	+	+	+	−	+	−	−	+	NA
Ranson et al. [66]	+	+	+	+	+	+	+	+	+	+	+	+
Rashidian et al. [67]	+	+	+	+	+	+	+	+	+	+	+	NA
Rezaei-Hemami et al. [68]	+	−	+	−	+	+	+	+	NA	+	+	NA
Saygili et al. [69]	+	+	+	+	+	+	+	+	NA	+	−	NA
Shamshiri et al. [70]	+	+	+	+	+	+	+	+	+	−	−	+
Sharma et al. [71]	+	+	+	+	+	+	+	+	+	+	−	NA
Shlomai et al. [72]	+	+	+	+	+	+	+	+	NA	+	NA	−
Shmueli et al. [73]	+	+	+	+	+	+	+	+	+	−	+	+
Sladkevicius et al. [74]	+	+	+	+	+	+	+	+	−	+	−	NA
Verguet et al. [75]	−	+	+	+	+	−	+	+	−	+	+	+
Vijayaraghavan et al. [76]	+	+	+	+	+	+	+	+	+	+	+	NA
Vijayaraghavan et al. [77]	+	+	+	+	+	−	+	+	+	+	+	NA
Wilcox et al. [78]	+	+	+	+	+	−	+	+	+	+	+	NA
Yarahmadi et al. [79]	+	+	+	+	+	−	+	+	+	+	+	NA
Yosefy et al. [80]	+	+	+	+	+	−	+	+	+	+	+	+
Yosefy et al. [81]	+	+	+	+	+	−	−	−	+	+	+	+
*No. of missed items (% applicable items)*	1 (2)	8 (13)	0 (0)	3 (5)	1 (2)	21 (34)	4 (7)	15 (25)	14 (25)	9 (15)	15 (27)	5 (19)

Author (References)	Resources and costs	Currency and price Conversion	Model description	Assumptions	Analytic methods	Result parameters	ICER	Uncertainty	Heterogeneity	Findings and limitations	Funding source	Conflict of interest	Score (% of applicable items)
Adibi et al. [21]	+	+	+	+	+	+	+	+	NA	+	+	+	22 (100)
Al Abri et al. [22]	+	+	+	−	+	+	+	+	−	+	+	+	20 (83)
Al‐Qudah et al. [23]	+	+	−	+	+	+	NA	+	−	+	+	+	18 (86)
Assanelli et al. [24]	+	+	−	−	+	+	+	+	NA	+	+	+	17 (77)
Balicer et al. [25]	+	−	−	+	−	−	NA	−	+	+	−	−	12 (55)
Barfar et al. [26]	+	+	−	+	+	+	+	+	NA	+	+	+	21 (95)
Carvalho et al. [27]	+	−	+	+	+	+	+	+	NA	+	+	+	18 (82)
Chodick et al. [28]	+	−	+	+	+	+	+	+	−	+	−	−	19 (83)
Chodick et al. [29]	+	−	+	+	−	+	+	−	+	+	+	−	19 (79)
Chowers [30]	+	+	+	+	+	+	+	+	NA	+	−	+	20 (87)
Devine [31]	+	−	−	+	+	+	+	+	−	+	+	+	20 (87)
El-Dahiyat [32]	+	−	NA	+	−	+	NA	−	NA	+	+	+	12 (67)
El-Ghitany [33]	−	−	−	−	−	+	−	−	−	−	+	+	8 (35)
Eltabbakh et al. [34]	+	−	−	+	−	+	+	−	−	+	−	+	14 (58)
Gamaoun et al. [35]	−	+	+	+	−	+	+	−	+	+	−	+	17 (74)
Ginsberg et al. [36]	+	+	−	+	−	+	+	+	NA	+	−	−	17 (77)
Ginsberg et al. [37]	+	+	+	+	+	+	+	+	NA	+	−	+	18 (78)
Ginsberg et al. [38]	+	+	+	+	+	+	+	+	NA	+	+	+	21 (91)
Ginsberg et al. [39]	+	+	+	+	+	−	+	+	+	+	−	−	20 (83)
Ginsberg et al. [40]	+	+	−	+	+	+	+	+	−	+	−	+	18 (82)
Haghighat et al. [41]	+	+	+	+	+	+	+	+	+	+	+	+	24 (100)
Hamdani et al. [42]	+	+	−	+	−	+	+	−	NA	+	+	+	19 (86)
Howard et al. [43]	+	+	−	+	+	+	+	+	−	+	+	+	21 (88)
Hussain et al. [44]	+	+	+	+	+	+	+	+	NA	+	+	+	23 (100)
Javadinasab et al. [45]	+	+	+	+	+	+	+	+	+	+	−	−	21 (88)
Javanbakht [46]	+	+	+	+	+	+	−	−	−	+	+	+	17 (77)
Kashi et al. [47]	+	+	+	+	−	+	+	+	NA	+	+	+	21 (91)
Khneisser et al. [48]	+	−	NA	−	−	+	NA	−	NA	+	+	+	10 (50)
Khneisser et al. [49]	+	−	NA	−	−	+	NA	−	NA	+	+	+	11 (55)
Kim et al. [50]	+	+	+	+	+	+	+	+	+	+	+	−	23 (96)
Kim et al. [51]	+	+	+	+	+	+	+	+	+	+	+	+	23 (100)
Koren et al. [52]	+	+	−	−	−	+	−	−	NA	+	+	+	13 (59)
Lahiri et al. [53]	+	−	+	+	−	+	+	+	−	+	+	−	18 (82)
Leshno et al. [54]	+	+	+	+	+	−	+	+	NA	−	+	−	17 (77)
Lim et al. [55]	+	−	+	+	+	+	−	+	−	+	+	+	18 (78)
Lohse et al. [56]	+	−	+	+	−	+	+	−	NA	+	+	+	16 (70)
Madae’en et al. [57]	+	−	+	+	+	+	+	+	+	+	+	+	22 (96)
Marseille et al. [58]	+	+	+	+	+	+	+	+	NA	+	+	+	20 (87)
Mason et al. [59]	+	+	+	+	+	+	+	+	−	+	+	+	21 (91)
Messoudi et al. [60]	+	+	+	+	+	+	+	+	+	+	+	+	23 (100)
Mostafa et al. [61]	+	+	+	+	+	+	+	+	−	+	+	+	23 (96)
Mostafa et al. [62]	+	+	+	+	+	+	+	+	−	+	+	+	23 (96)
Nahvijou et al. [63]	+	+	+	+	+	+	+	+	−	+	+	+	23 (96)
Okem et al. [64]	+	+	+	+	−	+	+	−	NA	+	−	−	16 (73)
Ornoy et al. [65]	+	+	−	+	−	+	−	−	NA	+	+	+	15 (68)
Ranson et al. [66]	+	+	−	+	−	+	+	−	NA	+	−	−	18 (78)
Rashidian et al. [67]	+	+	−	+	+	+	+	+	+	+	+	+	22 (96)
Rezaei-Hemami et al. [68]	+	−	−	−	+	+	−	−	−	+	+	+	14 (64)
Saygili et al. [69]	+	−	−	+	−	+	+	−	NA	+	−	+	15 (71)
Shamshiri et al. [70]	+	+	+	+	+	+	+	+	NA	+	+	−	20 (87)
Sharma et al. [71]	−	+	+	+	−	+	+	−	+	+	+	+	19 (83)
Shlomai et al. [72]	+	−	+	+	+	+	+	+	−	+	+	+	19 (86)
Shmueli et al. [73]	+	+	+	+	+	+	+	+	NA	+	+	−	21 (91)
Sladkevicius et al. [74]	+	+	+	+	+	+	+	+	NA	+	+	+	20 (91)
Verguet et al. [75]	−	−	+	+	+	+	+	+	−	+	−	+	17 (71)
Vijayaraghavan et al. [76]	+	−	+	+	+	+	+	+	NA	+	−	−	19 (86)
Vijayaraghavan et al. [77]	+	−	−	+	+	+	+	+	NA	+	+	+	19 (86)
Wilcox et al. [78]	+	+	−	+	+	+	+	+	−	+	+	−	19 (83)
Yarahmadi et al. [79]	−	−	NA	−	−	+	−	−	NA	+	−	−	12 (57)
Yosefy et al. [80]	+	+	−	+	−	+	−	+	−	+	−	+	18 (75)
Yosefy et al. [81]	+	+	−	−	+	+	+	−	−	+	+	−	16 (67)
*No. of missed items (% applicable items)*	5 (8)	21 (34)	21 (37)	9 (15)	21 (34)	3 (5)	8 (14)	20 (33)	21 (64)	2 (3)	17 (28)	17 (28)	

*ICER* incremental cost-effectiveness ratio, *NA* not applicable, (+): reported, (–): not reported

Against applicable CHEERS items of each study, 21 studies (37%) did not explicitly mention the modeling approach and did not provide the model structure (item 15). Data on the quantities and unit costs of the resources used, the methods of currency conversion and the exchange rates (item 14), the description of the analytical methods supporting the evaluation (item 17), and the perspective (item 6) were missed in 21 studies (34%). Similarly, 21 studies (64%) did not specify whether they dealt with heterogeneity (item 21), as shown in Table 4.

## Discussion

This review identified a heterogeneous body of literature on economic evaluations of different public health interventions in MENA countries, dominated by several screening programs (n = 36; 59%). In addition, the number of published studies has been growing over time, particularly after 2013, and appears to be generated only in a few countries: Israel, Iran, Egypt, and Pakistan, contributing to 38 studies or 62% of the economic evaluation studies across all MENA countries. However, the number of published economic evaluation studies identified in this review remains very low and might not truly reflect the research efforts in the region. In this case, the actual number of economic evaluation studies on public health interventions might be higher in this region as studies are more likely to be written in local languages for submission to local authorities. Also, unlike economic evaluations of pharmaceutical products and vaccines, economic evaluation studies on public health interventions do not attract industry sponsorship; therefore, the chance of their publication is limited.

Consequently, this review found that the number of economic evaluation studies on public health interventions was lower than economic evaluation studies on diagnostic and therapeutic interventions (69 studies) [19] but higher than those on vaccines (46 studies) [18] in the MENA region. Besides, the low number of studies on public health intervention might be partly related to low public investment in public health interventions coupled with an industry-low interest in such interventions, considering that industry has contributed a large portion (32%) of the funding for economic evaluation studies in other regions [83]. The low number of innovative interventions in public health areas might also play a role. In addition, it could be explained by the fact that public health policy decision-making in many countries in the MENA region is not currently guided by the economic evaluation evidence and that there is a lack of region-specific data on important parameters such as epidemiological data and public health program effectiveness that could facilitate the conduct of economic evaluation studies. Furthermore, barriers to conducting economic evaluations still exist in this region. These barriers have been discussed elsewhere in previous studies [6, 18, 19, 84–86].

Moreover, several gaps were identified in this review. *First* is a geographical gap; there was a complete absence of economic evaluation studies from several high-income countries (i.e., Bahrain, Kuwait, Qatar, Saudi Arabia and UAE), middle-income countries (i.e., Djibouti, Iraq, Mauritania and Palestine), and low-income countries (Sudan and Yemen). In these countries, HE and HTA studies are not mandatory in decision-making [6, 18, 19, 84, 85]. Meanwhile, these countries share some common features: the lack of HTA institutionalization, the absence of national HE guidelines, the lack of HE and HTA graduate and post-graduate academic programs, the limited technical capacity to conduct economic evaluation studies, and the fragility of UHC programs [6, 18, 19, 84, 85]. Another possible explanation for the low number of economic evaluation studies in high-income and low-income countries in MENA is that high-income countries (Gulf States) have sufficient financial resources to adopt expensive interventions, while public health interventions in low-income countries are mainly funded by donors and international agencies. Based on the results of this study, 19 studies (31%) and 11 studies (18%) were conducted in high- and low-income countries, respectively. On the other hand, 29 studies (48%) were conducted in middle-income countries. These middle-income countries have faced many challenges in adopting new public health interventions under finite resources; hence, HE and HTA evidence are greatly needed to support policy decision-making. As such, an increase in the number of economic evaluation studies from these countries shortly can be expected.

While this review found that most of the identified studies were of good quality, other factors influenced the transferability or generalizability of economic evaluation results across jurisdictions. These included epidemiological data, resource use, unit costs, utility value, health care delivery pattern, and effectiveness of public health interventions. As the MENA region comprises different countries in different geographies and income levels, these parameters might not be similar across all countries. For high-income countries in the region, this review identified 19 studies. Of which, 18 were from Israel, which might limit the generalizability or transferability to other high-income countries in the region (Gulf States) due to differences in the above factors. The researchers also identified 29 studies from middle-income countries, out of which 20 studies came from Egypt, Iran, and Pakistan. In this case, the studies identified in these middle-income countries can be generalized to other countries within the same income group in the region.

Furthermore, 11 studies from low-income countries were identified. Many of them were conducted as multi-country studies, indicating the possibility of transferring their results across low-income countries. Also, it is important to note that the transferability of such findings could not be applied solely to all countries in the region. Even within the same income group in the MENA region, similarities in terms of epidemiology data, disease burden, and patterns of health care delivery should be considered when transferring economic evaluation evidence. Generally, they can be transferred to close similar countries. For example, the findings of high-quality studies conducted in Pakistan can be transferred to Afghanistan and vice versa.

Similarly, high-quality studies conducted in Algeria, Morocco, and Tunisia can be generalized among these countries. The same can be applied to Iran, Iraq, Jordan, Lebanon, and Turkey. Although there was a limited number of economic evaluation studies in the region, the authors believe that transferability could be generally made across the middle- and low-income countries in the region, as they are less heterogeneous in terms of epidemiological parameters, patterns of healthcare delivery, unit cost, and utility values. Therefore, economic evaluation evidence is suitable to inform policy decision-making in similar settings but not in the wider range of the region. However, for decision-makers, careful consideration is required when transferring and adopting economic evaluation results from studies conducted outside their countries, and more economic evaluation studies are warranted to support evidence-based policy decision-making.

*Second*, there is also a gap in research quality: although the quality of the included studies was generally good [44 studies (72%) achieved scores of 75% or over], there is room to improve the overall quality of HE studies in this region*.* Utilizing locally-generated epidemiological data, unit cost of health service, effectiveness and utility data, model calibration and validation, dealing with skewed and missing data, and testing the robustness of results are all warranted. It is also required to explicitly describe the modeling approach and provide the model structure, mention the currency conversion methods and the exchange rates, report the perspective, and deal with heterogeneity. It is noteworthy that the number and quality of economic evaluation studies conducted in countries where HE academic programs, national HE guidelines, and HTA agencies exist (i.e., Egypt, Iran, Israel, Turkey, and Tunisia) tend to be much better than studies performed in countries where HE academic programs and guidelines are still inexistent.

As revealed by this study’s results, more than half of the studies conducted in the region (33 studies; 54%) were performed in 3 countries (i.e., Israel, Iran, and Egypt), where HTA and HE had formal roles in the decision-making process [18, 19]. Besides, the researchers observed that studies performed by health economists and public health professionals tended to be of better quality than studies performed by clinicians. Furthermore, the researchers noticed the relationship between the year of publication and the overall quality of the study. Studies published since 2015 tended to be of better quality than the previous studies. It may be due to the development of standards and guidelines aimed to ensure the quality of reporting in the HE field.

This study’s results showed that the economic evaluation studies were conducted for the following diseases: infectious diseases (21 studies; 34%), cancers (13 studies; 21%), genetic disorders (nine studies; 15%), CVDs (seven studies; 12%), and other disease domains including maternal diseases, back pain, malnutrition, gestational diabetes mellitus, and mental diseases (11 studies; 18%). The observed research output did not adequately reflect the current and upcoming disease burden and risk factors trends in the MENA region. Currently, several MENA countries have some of the highest rates of NCD-risk factors, such as high blood pressure, obesity, physical inactivity, tobacco use, and high intake of salt, sugar and fats [15, 87]. Likewise, many diseases, such as CVDs, cancers, diabetes, and chronic lung and kidney diseases, are serious public health issues representing a significant disease burden in the MENA region. These risk factors and diseases place enormous pressures on the health system and resources; therefore, they require significant policy attention. For example, the MENA region experienced the highest global increase in the prevalence of diabetes mellitus in 2019 (12.2% of the adult population aged 20–79 years) and is expected to witness the second-highest increase (96%) in this prevalence between 2019 and 2045 compared to other parts of the world [88]*.* Similarly, a recently published review article indicated that the overall estimated pooled prevalence of hypertension in MENA was 26% and is estimated to double by 2025 [89]*.* Another recent study indicated that smoking prevalence was 62.7% and 27.5% among adult men and women in some MENA countries, respectively. These rates ranked among the highest worldwide [90].

On the other hand, MENA countries have witnessed many infectious disease outbreaks, such as polio outbreaks (Afghanistan, Pakistan, and Syria) and cholera outbreaks (Iraq and Yemen). Highly pathogenic and serious viral infections like hepatitis B and hepatitis C are also still an important risk of morbidity and mortality and pose a real threat to some MENA countries like Egypt. Similarly, malaria, hepatitis A virus, Chikungunya, dengue fever, cholera, diphtheria, Leishmaniasis, measles, and Rift Valley fever constitute major health, social, and economic challenges for several MENA countries [12, 91].

By the end of the study identification process, the researchers could find only one economic evaluation study on public health interventions to control and prevent COVID-19 [72]. Given the growing concern about the economic impact and value of such interventions (lockdowns, border closures, screening of suspected cases, tracing and isolating symptomatic individuals and their contacts, quarantine, personal protective equipment, and social distancing), the researchers may expect that the number of economic evaluation studies on COVID-19 interventions will increase globally, including the MENA region as these interventions have substantial economic and social consequences. In addition, a recent systematic review of these interventions suggested that screening and social distancing have been cost-effective in preventing and controlling COVID-19 over a long-time horizon. However, the evidence remains inconclusive and too heterogeneous to provide firm conclusions regarding the costs of the interventions [92]. In emergencies or epidemics such as COVID-19, it is not justified to delay policy decisions in priority areas due to the immaturity of scientific evidence (e.g., economic evaluation studies). Waiting for a better evidence base to judge the true value of potentially beneficial interventions increases the risk of infection spreading, which could have huge social and economic implications. However, decisions have to comply with the basic principles of what is considered good evidence-based decision-making practices as much as possible, while at the same time, additional data must be collected, generated, and evaluated; this strategy is called "coverage with evidence development". These decisions should be reviewed regularly as new evidence emerges [93].

Notably, there was a complete absence of economic evaluation studies on interventions targeting specific realms, such as physical inactivity, alcohol drinking, occupational health, and mental health. Previous global systematic reviews on economic evaluations of occupational health interventions also revealed no single study from the MENA region and a very limited number from other regions, indicating a great need for further research in these domains [94, 95]. Another study covering some MENA countries indicated that occupational health research is neglected, although occupational injuries have been reported to be high in these countries [91]. One potential reason for this negligence of occupational health research in MENA countries is the lack of experts or awareness about the importance of this discipline. Likewise, the lack of economic evaluation studies on mental health may result from the absence of governments’ spending on mental health, insufficient capacity, and the absence of public health interventions in this field. Following the same pattern, the lack of economic evaluation studies on interventions targeting alcohol drinking may be caused by the fact that almost all MENA countries are dominated by Muslim populations, where drinking alcohol is religiously forbidden; therefore, these countries may not bear the same burden experienced in other regions. Meanwhile, the lack of economic evaluation studies on interventions promoting physical activity despite the region's high prevalence of diabetes, obesity, and NCDs cannot be justified.

Moreover, it is noteworthy that the researchers observed that most public health interventions were significantly cost-effective or even cost-saving. Even low-intensity public health interventions can have strong positive effects on the population’s health in less developed countries and may positively impact the possibility of reducing poverty and boosting economic indicators and living standards. In this sense, a public health system that responds to the health need is a facilitator of human development since components of human capital, such as health and education, are positively correlated with the quality of the public health system [96]. It is also evident that healthier countries tend to be wealthier than countries with lower health status, a relationship known as the “Preston curve” [97]. In addition, public health interventions may generate wider economic consequences than those related to the healthcare sector alone. Investing in public health interventions, therefore, extends to many other sectors, such as education, manufacturing, commerce, tourism, and transportation [98]. Paradoxically, despite proven benefits and long-term returns on investment, public health is frequently considered a politically easy target for budget cuts, as reported in some MENA countries and elsewhere [98].

Further, the findings of this review reflect a misperception of the role of public health in communities. In this region, as in other parts of the world, the political nature of policy-making processes is apparent where policy-makers tend to advocate for curative interventions and invest in building large hospitals and medical cities as they perceive it as the best way to foster health and well‑being. However, the healthcare system has other tasks with prevention, health promotion, health education, and early disease detection even before one becomes ill and requires a hospital visit [99]. Thus, the focus must shift to upgrading the healthcare system to become more proactive, comprehensive, and integrated. As such, health issues can be addressed earlier, and quality healthcare services can be accessed once needed [100]. Guided by the WHO resolution on HTA in support of UHC [101] and motivated by the challenges of rising and escalating healthcare costs under the economic pressure these countries are suffering from, more serious local initiatives are required to establish a healthcare prioritization system (including HTA) transparently and legitimately, to ensure the sustainability of healthcare systems and to promote equitable, efficient, and high-quality healthcare services. In addition, worth mentioning that MENA countries began to implement effective interventions and policies to curb NCDs risk factors [13], yet these responses have been slow and appear not to be fully aligned with each country's social, economic, and health situations [11]. Further, these policies have largely focused on clinical and curative activities rather than preventive, educative, and promotive services.

On the other side, disease burden is commonly seen as an important criterion for low- and middle-income countries (LMICs) in national decision-making to prioritize research directions [102–104]. While it is indicative of the potential scale of the population that would benefit from a healthcare intervention, understanding the value of interventions that target high disease burdens requires information about the cost of those interventions and the opportunity cost of funding them. It can be operationalized using a threshold reflecting health opportunity cost. Country-specific thresholds reflecting the healthcare system, local priorities, local preferences, and ability to pay are also needed in the MENA region (see Table 3). More details and discussions were also provided in previous reviews [6, 18, 19, 84–86].

This study is the first systematic review of economic evaluations of public health intervention in the MENA region, highlighting under-studied disease areas to fill these clear gaps. Although the strengths of this study lie in its rigorous and systematic search strategy, broad time horizon (database inception onwards), geographic coverage of all 26-MENA countries, and the quality assessment of included studies, this study has some limitations. First, it has been difficult to include all that constitutes a ‘public health intervention’ due to terminology diffusion. As such, the researchers had limited the search to the abovementioned terms. Second, the search was limited to studies published in English identified from the two main databases: PubMed and Scopus. Hence, this review did not include technical reports, HE, HTA, and other grey literature written in local languages. Concerning this, it can be argued that government institutions may perform more economic evaluation studies, but as in some other regions, they may not choose to publish their results due to the political environment in which the decision-making process remains largely secretive with limited transparency [105]. However, the full impact of such exclusions on this study’s results still requires further identification. Therefore, further studies covering more databases (Embase, Cochrane, and Cost-Effectiveness Analysis Registry, and others), unpublished studies, and studies published in local languages are warranted. Third, this review did not include HE studies that evaluated vaccines, diagnostic tools, pharmaceutical products, or other therapeutic interventions. These studies were evaluated separately to ensure harmony and consistency of compared studies, thus, gaining more insight into the specific characteristics of each group of interventions [18, 19]. Lastly, focusing on full HE studies, partial economic evaluations such as cost-of-illness studies were excluded from this review.

## Conclusions

The number of economic evaluation studies on public health interventions conducted in MENA countries has increased in the past years; however, this number was still very limited, indicating that economic evaluation evidence is not widely used to guide public health decisions in the MENA region. Geographic gaps across countries in the region were also identified concerning the number of economic evaluation studies. In addition, this review found that the number of economic evaluation studies did not align with disease domains with the highest burden and the most prevalent risk factors in some countries. It suggested that MENA governments should prioritize the disease domains and innovative interventions when funding economic evaluation studies to reflect the health burden in the region better. Although the overall quality of the reviewed studies was good—more than two-thirds of them were of high to excellent quality—and were potentially useful for policy decision-making, the limited number of studies in high- and low-income countries and the transferability issues across jurisdictions since MENA region consisted of heterogeneous countries suggested that the existing economic evaluation evidence might not be sufficient to informed policy decision-making in the wider range of the region. The commitment to adopting economic evaluation evidence for public health policy decision-making and developing economic evaluation evidence on major disease burdens are also clearly warranted for efficient public health resource allocation in the MENA region. To facilitate this, national HE guidelines and institutionalizing HTA policy should be established in all countries.

## Data Availability

The datasets generated and/or analyzed during the current study are available from the corresponding author upon reasonable request.

## References

[1] World Health Organization. Continuity and coordination of care: a practice brief to support implementation of the WHO Framework on integrated people-centred health services. 2018. https://apps.who.int/iris/bitstream/handle/10665/274628/9789241514033-eng.pdf. Accessed 19 Oct 2021.

[2] Alderwick H, Hutchings A, Briggs A, Mays N (2021). The impacts of collaboration between local health care and non-health care organizations and factors shaping how they work: a systematic review of reviews. BMC Public Health.

[3] Budreviciute A, Damiati S, Sabir DK, Onder K, Schuller-Goetzburg P, Plakys G (2020). Management and prevention strategies for non-communicable diseases (NCDs) and their risk factors. Front Public Health.

[4] Reeves P, Edmunds K, Searles A, Wiggers J (2019). Economic evaluations of public health implementation-interventions: a systematic review and guideline for practice. Public Health.

[5] Mueller D, Tivey D, Croce D (2017). Health-technology assessment: its role in strengthening health systems in developing countries. South Afr J Pub Health.

[6] Fasseeh A, Karam R, Jameleddine M, George M, Kristensen FB, Al-Rabayah AA (2020). Implementation of Health Technology Assessment in the Middle East and North Africa: comparison between the current and preferred status. Front Pharmacol.

[7] World Health Organization. A vision for primary health care in the 21st century: towards universal health coverage and the Sustainable Development Goals. Geneva: World Health Organization and the United Nations Children’s Fund (UNICEF): World Health Organization. 2018. https://apps.who.int/iris/bitstream/handle/10665/328065/WHO-HIS-SDS-2018.15-eng.pdf. Accessed 19 Oct 2021.

[8] Wang DWL (2020). Priority-setting and the right to health: synergies and tensions on the path to universal health coverage. Hum Rights Law Rev.

[9] Bcheraoui CE, Charara R, Khalil I, Moradi-Lakeh M, Afshin A, Collison M (2018). Danger ahead: the burden of diseases, injuries, and risk factors in the Eastern Mediterranean Region, 1990–2015. Int J Public Health.

[10] GBD 2019 Diseases and Injuries Collaborators. Global burden of 369 diseases and injuries in 204 countries and territories, 1990–2019: a systematic analysis for the Global Burden of Disease Study 2019. Lancet. 2020;396(10258):1204–22. 10.1016/S0140-6736(20)30925-9 PMC7567026 33069326

[11] Aggarwal A, Patel P, Lewison G, Ekzayez A, Coutts A, Fouad FM (2020). The profile of non-communicable disease (NCD) research in the Middle East and North Africa (MENA) region: analyzing the NCD burden, research outputs and international research collaboration. PLoS ONE.

[12] Mostafavi E, Ghasemian A, Abdinasir A, Nematollahi Mahani SA, Rawaf S, Salehi Vaziri M, et al. Emerging and re-emerging infectious diseases in the WHO Eastern Mediterranean Region, 2001–2018. Int J Health Policy Manag. 2021. 10.34172/ijhpm.2021.13 PMC9808364 33904695

[13] El-Saharty S, Kaneda T, Liu AC. Tackling noncommunicable diseases in the Arab Region. Laher I, editor. Springer, Cham; 2021. p. 789–836.

[14] World Health Organization. Burden of noncommunicable diseases in the Eastern Mediterranean Region. WHO Regional Office for Eastern Mediterranean Region; Cairo. 2015. http://www.emro.who.int/noncommunicable-diseases/publications/burden-of-noncommunicable-diseases-in-the-eastern-mediterranean-region.html. Accessed 30 April 2022.

[15] Fikri M, Hammerich A (2018). Scaling up action on the prevention and control of noncommunicable diseases in the WHO Eastern Mediterranean Region. East Mediterr Health J.

[16] Moher D, Liberati A, Tetzlaff J, Altman DG (2009). Preferred reporting items for systematic reviews and meta-analyses: the PRISMA statement. PLoS Med.

[17] Abed GT, Davoodi HR. Challenges of growth and globalization in the Middle East and North Africa. International Monetary Fund; 2003.

[18] Nagi MA, Luangsinsiri C, Thavorncharoensap M (2021). A systematic review of economic evaluations of vaccines in Middle East and North Africa countries: is existing evidence good enough to support policy decision-making?. Expert Rev Pharmacoecon Outcomes Res.

[19] Nagi MA, Dewi PEN, Thavorncharoensap M, Sangroongruangsri S. A systematic review on economic evaluation studies of diagnostic and therapeutic interventions in the Middle East and North Africa. Appl Health Econ Health Policy. 2021. 10.1007/s40258-021-00703-y 34931297

[20] Husereau D, Drummond M, Petrou S, Carswell C, Moher D, Greenberg D (2013). Consolidated health economic evaluation reporting standards (CHEERS)—explanation and elaboration: a report of the ISPOR health economic evaluation publication guidelines good reporting practices task force. Value Health.

[21] Adibi P, Rezailashkajani M, Roshandel D, Behrouz N, Ansari S, Somi MH (2004). An economic analysis of premarriage prevention of hepatitis B transmission in Iran. BMC Infect Dis.

[22] Al Abri S, Kowada A, Yaqoubi F, Al Khalili S, Ndunda N, Petersen E (2020). Cost-effectiveness of IGRA/QFT-Plus for TB screening of migrants in Oman. Int J Infect Dis.

[23] Al-Qudah RA, Al-Badriyeh D, Al-Ali FM, Altawalbeh SM, Basheti IA (2020). Cost-benefit analysis of clinical pharmacist intervention in preventing adverse drug events in the general chronic diseases outpatients. J Eval Clin Pract.

[24] Assanelli D, Levaggi R, Carré F, Sharma S, Deligiannis A, Mellwig KP (2015). Cost-effectiveness of pre-participation screening of athletes with ECG in Europe and Algeria. Intern Emerg Med.

[25] Balicer RD, Huerta M, Davidovitch N, Grotto I (2005). Cost-benefit of stockpiling drugs for influenza pandemic. Emerg Infect Dis.

[26] Barfar E, Rashidian A, Hosseini H, Nosratnejad S, Barooti E, Zendehdel K (2014). Cost-effectiveness of mammography screening for breast cancer in a low socioeconomic group of Iranian women. Arch Iran Med.

[27] Carvalho N, Salehi AS, Goldie SJ (2013). National and sub-national analysis of the health benefits and cost-effectiveness of strategies to reduce maternal mortality in Afghanistan. Health Policy Plan.

[28] Chodick G, Ashkenazi S, Livni G, Lerman Y (2005). Cost-effectiveness of varicella vaccination of healthcare workers. Vaccine.

[29] Chodick G, Lerman Y, Wood F, Aloni H, Peled T, Ashkenazi S (2002). Cost-utility analysis of hepatitis A prevention among health-care workers in Israel. J Occup Environ Med.

[30] Chowers M, Shavit O (2017). Economic evaluation of universal prenatal HIV screening compared with current 'at risk' policy in a very low prevalence country. Sex Transm Infect.

[31] Devine A, Howes RE, Price DJ, Moore KA, Ley B, Simpson JA (2020). Cost-effectiveness analysis of sex-stratified plasmodium vivax treatment strategies using available G6PD diagnostics to accelerate access to radical cure. Am J Trop Med Hyg.

[32] El-Dahiyat F (2017). Pharmacoeconomic evidence and policies to promote use of generic medicines in Jordan. Pharm Policy Law.

[33] El-Ghitany EM (2019). Cost-effectiveness of EGCRISC application versus hepatitis C virus mass screening in Egypt. J Infect Public Health.

[34] Eltabbakh M, Zaghla H, Abdel-Razek W, Elshinnawy H, Ezzat S, Gomaa A (2015). Utility and cost-effectiveness of screening for hepatocellular carcinoma in a resource-limited setting. Med Oncol.

[35] Gamaoun R (2018). National cervical cancer prevention program in the Arab States: strategies and cost-minimization study of the Tunisian case. Vaccine.

[36] Ginsberg G, Tulchinsky T, Filon D, Goldfarb A, Abramov L, Rachmilevitz EA (1998). Cost-benefit analysis of a national thalassaemia prevention programme in Israel. J Med Screen.

[37] Ginsberg GM (2013). Cost-utility analysis of interventions to reduce the burden of cervical cancer in Israel. Vaccine.

[38] Ginsberg GM, Chemtob D (2020). Cost utility analysis of HIV pre exposure prophylaxis among men who have sex with men in Israel. BMC Public Health.

[39] Ginsberg GM, Fisher M, Ben-Shahar I, Bornstein J (2007). Cost-utility analysis of vaccination against HPV in Israel. Vaccine.

[40] Ginsberg GM, Rosenberg E (2012). Economic effects of interventions to reduce obesity in Israel. Isr J Health Policy Res.

[41] Haghighat S, Akbari ME, Yavari P, Javanbakht M, Ghaffari S (2016). Cost-effectiveness of three rounds of mammography breast cancer screening in Iranian Women. Iran J Cancer Prev.

[42] Hamdani SU, Huma ZE, Rahman A, Wang D, Chen T, van Ommeren M (2020). Cost-effectiveness of WHO Problem Management Plus for adults with mood and anxiety disorders in a post-conflict area of Pakistan: randomised controlled trial. Br J Psychiatry.

[43] Howard N, Guinness L, Rowland M, Durrani N, Hansen KS (2017). Cost-effectiveness of adding indoor residual spraying to case management in Afghan refugee settlements in Northwest Pakistan during a prolonged malaria epidemic. PLoS Negl Trop Dis.

[44] Hussain H, Mori AT, Khan AJ, Khowaja S, Creswell J, Tylleskar T (2019). The cost-effectiveness of incentive-based active case finding for tuberculosis (TB) control in the private sector Karachi, Pakistan. BMC Health Serv Res.

[45] Javadinasab H, Daroudi R, Salimzadeh H, Delavari A, Vezvaie P, Malekzadeh R (2017). Cost-effectiveness of screening colonoscopy in Iranian high risk population. Arch Iran Med.

[46] Javanbakht M, Jamshidi AR, Baradaran HR, Mohammadi Z, Mashayekhi A, Shokraneh F (2018). Estimation and prediction of avoidable health care costs of cardiovascular diseases and type 2 diabetes through adequate dairy food consumption: a systematic review and micro simulation modeling study. Arch Iran Med.

[47] Kashi B, Godin CM, Kurzawa ZA, Verney AMJ, Busch-Hallen JF, De-Regil LM (2019). Multiple micronutrient supplements are more cost-effective than iron and folic acid: modeling results from 3 high-burden Asian countries. J Nutr.

[48] Khneisser I, Adib S, Assaad S, Megarbane A, Karam P (2015). Cost-benefit analysis: newborn screening for inborn errors of metabolism in Lebanon. J Med Screen.

[49] Khneisser I, Adib SM, Loiselet J, Megarbane A (2007). Cost-benefit analysis of G6PD screening in Lebanese newborn males. J Med Liban.

[50] Kim DD, Hutton DW, Raouf AA, Salama M, Hablas A, Seifeldin IA (2015). Cost-effectiveness model for hepatitis C screening and treatment: implications for Egypt and other countries with high prevalence. Glob Public Health.

[51] Kim JJ, Sharma M, O'Shea M, Sweet S, Diaz M, Sancho-Garnier H (2013). Model-based impact and cost-effectiveness of cervical cancer prevention in the Extended Middle East and North Africa (EMENA). Vaccine.

[52] Koren A, Profeta L, Zalman L, Palmor H, Levin C, Zamir RB (2014). Prevention of β Thalassemia in Northern Israel—a cost-benefit analysis. Mediterr J Hematol Infect Dis.

[53] Lahiri S, Markkanen P, Levenstein C (2005). The cost effectiveness of occupational health interventions: preventing occupational back pain. Am J Ind Med.

[54] Leshno M, Halpern Z, Arber N (2003). Cost-effectiveness of colorectal cancer screening in the average risk population. Health Care Manag Sci.

[55] Lim AG, Walker JG, Mafirakureva N, Khalid GG, Qureshi H, Mahmood H (2020). Effects and cost of different strategies to eliminate hepatitis C virus transmission in Pakistan: a modelling analysis. Lancet Glob Health.

[56] Lohse N, Marseille E, Kahn JG (2011). Development of a model to assess the cost-effectiveness of gestational diabetes mellitus screening and lifestyle change for the prevention of type 2 diabetes mellitus. Int J Gynaecol Obstet.

[57] Madae'en S, Obeidat N, Adeinat M (2020). Using cost-effectiveness analysis to support policy change: varenicline and nicotine replacement therapy for smoking cessation in Jordan. J Pharm Policy Pract.

[58] Marseille E, Lohse N, Jiwani A, Hod M, Seshiah V, Yajnik CS (2013). The cost-effectiveness of gestational diabetes screening including prevention of type 2 diabetes: application of a new model in India and Israel. J Matern Fetal Neonatal Med.

[59] Mason H, Shoaibi A, Ghandour R, O'Flaherty M, Capewell S, Khatib R (2014). A cost effectiveness analysis of salt reduction policies to reduce coronary heart disease in four Eastern Mediterranean countries. PLoS ONE.

[60] Messoudi W, Elmahi T, Nejjari C, Tachfouti N, Zidouh A, Saadani G (2019). Cervical cancer prevention in Morocco: a model-based cost-effectiveness analysis. J Med Econ.

[61] Mostafa A, El-Sayed MH, El Kassas M, Elhamamsy M, Elsisi GH (2019). Safety-engineered syringes: an intervention to decrease hepatitis C burden in developing countries—a cost-effectiveness analysis from Egypt. Value Health Reg Issues.

[62] Mostafa A, Elsisi GH (2019). A cost-effectiveness analysis of the use of safety-engineered syringes in reducing HBV, HCV, and HIV burden in Egypt. Expert Rev Med Devices.

[63] Nahvijou A, Daroudi R, Tahmasebi M, Amouzegar Hashemi F, Rezaei Hemami M, Akbari Sari A (2016). Cost-effectiveness of different cervical screening strategies in Islamic Republic of Iran: a middle-income country with a low incidence rate of cervical cancer. PLoS ONE.

[64] Ökem ZG, Örgül G, Kasnakoglu BT, Çakar M, Beksaç MS (2017). Economic analysis of prenatal screening strategies for Down syndrome in singleton pregnancies in Turkey. Eur J Obstet Gynecol Reprod Biol.

[65] Ornoy A, Spivak A (2019). Cost effectiveness of optimal treatment of ADHD in Israel: a suggestion for national policy. Health Econ Rev.

[66] Ranson MK, Jha P, Chaloupka FJ, Nguyen SN (2002). Global and regional estimates of the effectiveness and cost-effectiveness of price increases and other tobacco control policies. Nicotine Tob Res.

[67] Rashidian A, Alinia C, Majdzadeh R (2015). Cost-effectiveness analysis of health care waste treatment facilities in iran hospitals; a provider perspective. Iran J Public Health.

[68] Rezaei-Hemami M, Akbari-Sari A, Raiesi A, Vatandoost H, Majdzadeh R (2014). Cost effectiveness of malaria interventions from preelimination through elimination: a study in Iran. J Arthropod Borne Dis.

[69] Saygili M, Çelik Y (2019). An evaluation of the cost-effectiveness of the different palliative care models available to cancer patients in Turkey. Eur J Cancer Care (Engl).

[70] Shamshiri AR, Yarahmadi S, Forouzanfar MH, Haghdoost AA, Hamzehloo G, Holakouie NK (2012). Evaluation of current guthrie TSH cut-off point in Iran congenital hypothyroidism screening program: a cost-effectiveness analysis. Arch Iran Med.

[71] Sharma M, Seoud M, Kim JJ (2017). Cost-effectiveness of increasing cervical cancer screening coverage in the Middle East: an example from Lebanon. Vaccine.

[72] Shlomai A, Leshno A, Sklan EH, Leshno M. Modeling social distancing strategies to prevent SARS-CoV-2 spread in Israel: a cost-effectiveness analysis. Value Health. 2020. 10.1016/j.jval.2020.09.013 PMC7833124 33933228

[73] Shmueli A, Fraifeld S, Peretz T, Gutfeld O, Gips M, Sosna J (2013). Cost-effectiveness of baseline low-dose computed tomography screening for lung cancer: the Israeli experience. Value Health.

[74] Sladkevicius E, Pollitt RJ, Mgadmi A, Guest JF (2010). Cost effectiveness of establishing a neonatal screening programme for phenylketonuria in Libya. Appl Health Econ Health Policy.

[75] Verguet S, Stalcup M, Walsh JA (2013). Where to deploy pre-exposure prophylaxis (PrEP) in sub-Saharan Africa?. Sex Transm Infect.

[76] Vijayaraghavan M, Lievano F, Cairns L, Wolfson L, Nandy R, Ansari A (2006). Economic evaluation of measles catch-up and follow-up campaigns in Afghanistan in 2002 and 2003. Disasters.

[77] Vijayaraghavan M, Wallace A, Mirza IR, Kamadjeu R, Nandy R, Durry E (2012). Economic evaluation of a Child Health Days strategy to deliver multiple maternal and child health interventions in Somalia. J Infect Dis.

[78] Wilcox ML, Mason H, Fouad FM, Rastam S, al Ali R, Page TF (2015). Cost-effectiveness analysis of salt reduction policies to reduce coronary heart disease in Syria, 2010–2020. Int J Public Health.

[79] Yarahmadi S, Tabibi S, Alimohammadzadeh K, Ainy E, Gooya MM, Mojarrad M (2010). Cost-benefit and effectiveness of newborn screening of congenital hypothyroidism: findings from a national program in Iran. Int J Endocrinol Metab.

[80] Yosefy C, Ginsberg G, Viskoper R, Dicker D, Gavish D (2007). Cost-utility analysis of a national project to reduce hypertension in Israel. Cost Eff Resour Alloc.

[81] Yosefy C, Ginsberg GM, Dicker D, Viskoper JR, Tulchinsky TH, Leibovitz E (2003). Risk factor profile and achievement of treatment goals among hypertensive patients from the Israeli Blood Pressure Control (IBPC) program–initial cost utility analysis. Blood Press.

[82] Geng J, Yu H, Mao Y, Zhang P, Chen Y (2015). Cost effectiveness of dipeptidyl peptidase-4 inhibitors for type 2 diabetes. Pharmacoeconomics.

[83] Decimoni TC, Leandro R, Rozman LM, Craig D, Iglesias CP, Novaes HMD (2018). Systematic review of health economic evaluation studies developed in Brazil from 1980 to 2013. Front Public Health.

[84] Almazrou SH, Alaujan SS, Al-Aqeel SA (2021). Barriers and facilitators to conducting economic evaluation studies of Gulf Cooperation Council (GCC) countries: a survey of researchers. Health Res Policy Syst.

[85] Zrubka Z, Rashdan O, Gulácsi L (2020). Health economic publications from the Middle East and North Africa Region: a scoping review of the volume and methods of research. JQSH.

[86] Kim T, Sharma M, Teerawattananon Y, Oh C, Ong L, Hangoma P (2021). Addressing challenges in health technology assessment institutionalization for furtherance of universal health coverage through south-south knowledge exchange: lessons from Bhutan, Kenya, Thailand, and Zambia. Value Health Reg Issues.

[87] Fouad H, Latif N, Ingram R, Hammerich A (2018). Scaling up prevention and control of noncommunicable diseases in the WHO Eastern Mediterranean Region. East Mediterr Health J.

[88] El-Kebbi IM, Bidikian NH, Hneiny L, Nasrallah MP (2021). Epidemiology of type 2 diabetes in the Middle East and North Africa: Challenges and call for action. World J Diabetes.

[89] Khonsari NM, Shahrestanaki E, Ejtahed H-S, Djalalinia S, Sheidaei A, Hakak-Zargar B (2021). Long-term trends in hypertension prevalence, awareness, treatment, and control rate in the Middle East and North Africa: a systematic review and meta-analysis of 178 population-based studies. Curr Hypertens Rep.

[90] Nagi M, Riewpaiboon A, Thavorncharoensap M. Cost of premature mortality attributable to smoking in the Middle East and North Africa. East Mediterr Health J. 2021. 10.26719/emhj.21.028 34766323

[91] Sweileh WM, Zyoud SEH, Al-Jabi SW, Sawalha AF (2015). Public, environmental, and occupational health research activity in Arab countries: bibliometric, citation, and collaboration analysis. Arch Public Health.

[92] Rezapour A, Souresrafil A, Peighambari MM, Heidarali M, Tashakori-Miyanroudi M (2021). Economic evaluation of programs against COVID-19: a systematic review. Int J Surg (Lond Engl).

[93] Kaló Z, Németh B, Zemplényi A (2021). Can cost-effectiveness principles be ignored in urgent times?. J Comp Effect Res.

[94] Grimani K, Bergström G, Riaño-Casallas M, Aboagye E, Jensen I, Lohela-Karlsson M (2017). Economic evaluation of occupational safety and health interventions from the employer perspective: a systematic review. J Occup Environ Med.

[95] Do LA, Synnott PG, Ma S, Ollendorf DA (2021). Bridging the gap: aligning economic research with disease burden. BMJ Glob Health.

[96] Sharma R (2018). Health and economic growth: evidence from dynamic panel data of 143 years. PLoS ONE.

[97] Ghimire U (2020). The impact of health on wealth: empirical evidence.

[98] Masters R, Anwar E, Collins B, Cookson R, Capewell S (2017). Return on investment of public health interventions: a systematic review. J Epidemiol Community Health.

[99] Ross AM, de Saxe ZL (2020). Prevention, health promotion, and social work: aligning health and human service systems through a workforce for health. Am J Public Health.

[100] Islam QM (2021). Innovation in primary healthcare in the twenty-first century. J Health Manag.

[101] World Health Organization. World Health Assembly, 67. (2014). Health intervention and technology assessment in support of universal health coverage. 2014. https://apps.who.int/iris/handle/10665/162870. Accessed 29 Oct 2021.

[102] van der Putten IM, Evers SM, Deogaonkar R, Jit M, Hutubessy RC (2015). Stakeholders' perception on including broader economic impact of vaccines in economic evaluations in low and middle income countries: a mixed methods study. BMC Public Health.

[103] Youngkong S, Kapiriri L, Baltussen R (2009). Setting priorities for health interventions in developing countries: a review of empirical studies. Trop Med Int Health.

[104] Haider MS, Youngkong S, Thavorncharoensap M, Thokala P (2022). Priority setting of vaccine introduction in Bangladesh: a multicriteria decision analysis study. BMJ Open.

[105] Butt T, Liu GG, Kim DD, Neumann PJ (2019). Taking stock of cost-effectiveness analysis of healthcare in China. BMJ Glob Health.

